# COX6C expression driven by copy amplification of 8q22.2 regulates cell proliferation via mediation of mitosis by ROS-AMPK signaling in lung adenocarcinoma

**DOI:** 10.1038/s41419-024-06443-w

**Published:** 2024-01-19

**Authors:** Shuanghui Liu, Fanggui Shao, Yourong Wang, Yurui Zhang, Hongjia Yu, Ningxin Zhang, Lan He, Qingran Kong, Hao Jiang, Zhixiong Dong

**Affiliations:** 1 Zhejiang Provincial Key Laboratory of Medical Genetics, Key Laboratory of Laboratory Medicine, Ministry of Education, School of Laboratory Medicine and Life Science, Wenzhou Medical University, Wenzhou, Zhejiang 325035 P. R. China; 2 Laboratory Medicine Center, Department of Clinical Laboratory, Zhejiang Provincial People’s Hospital (Affiliated People’s Hospital, Hangzhou Medical College), Hangzhou, Zhejiang 310014 P. R. China; 3 Department of Laboratory Medicine, the First Affiliated Hospital of Wenzhou Medical University, Wenzhou, Zhejiang 325000 P. R. China; 4 Department of Clinical Laboratory, Key Laboratory of Clinical Laboratory Diagnosis and Translational Research of Zhejiang Province, the First Affiliated Hospital of Wenzhou Medical University, Wenzhou, Zhejiang 325000 P. R. China; 5 Department of clinical laboratory, Jinling Hospital, Affiliated Hospital of Medical School, Nanjing University, Nanjing, 210000 P. R. China; 6 School of Biomedical Science, Hunan University, Changsha, Hunan 410013 P. R. China; 7 Department of Biomedical Informatics, School of Life Sciences, Central South University, Changsha, Hunan 410013 P. R. China

**Keywords:** Non-small-cell lung cancer, Prognostic markers

## Abstract

Copy number variations (CNVs) play a vital role in regulating genes expression and tumorigenesis. We explored the copy number alterations in early-stage lung adenocarcinoma using high-throughput sequencing and nucleic acid flight mass spectrometry technology, and found that 8q22.1-22.2 is frequently amplified in lung adenocarcinoma tissues. *COX6C* localizes on the region and its expression is notably enhanced that driven by amplification in lung adenocarcinoma. Knockdown of COX6C significantly inhibits the cell proliferation, and induces S-G2/M cell cycle arrest, mitosis deficiency and apoptosis. Moreover, COX6C depletion causes a deficiency in mitochondrial fusion, and impairment of oxidative phosphorylation. Mechanistically, COX6C-induced mitochondrial deficiency stimulates ROS accumulation and activates AMPK pathway, then leading to abnormality in spindle formation and chromosome segregation, activating spindle assemble checkpoint, causing mitotic arrest, and ultimately inducing cell apoptosis. Collectively, we suggested that copy amplification-mediated COX6C upregulation might serves as a prospective biomarker for prognosis and targeting therapy in patients with lung adenocarcinoma.

## Introduction

Despite great advances in understanding of tumorigenesis, diagnosis, and therapeutic option, lung cancer remains one of the most common malignant cancers in the world and the leading cause of cancer-related death [1]. Lung adenocarcinoma (LUAD) is the main subtype accounting for ~40% of all cases of lung cancer. Patients with advanced LUAD were predicted worse prognosis, and the five years survival of them was estimated to be only about 15% [2]. Thus, identification and determination of new candidate genes involved in LUAD tumorigenesis would be beneficial to develop available and efficient strategy for diagnosis and therapy of LUAD.

DNA aberrations, including mutations, translocations, inversions and copy number variations (CNVs), are common and display crucial roles in promoting tumor progression. CNVs refer to a form of genomic structural variations that lead to abnormal gene copy numbers, including gene amplification, gain, loss and deletion. Previous studies found that tumor suppressors are often enriched in deleted CNVs, while oncogenes are enriched in gain or amplification CNVs [3]. For example, multiple known oncogenes, including *erb-b2 receptor tyrosine kinase 2* (*ERBB2*), *epidermal growth factor receptor* (*EGFR*), *Myc* and *CCND1*, are frequently amplified, and several known tumor suppressor genes, such as *cyclin dependent kinase inhibitor 2A* (*CDKN2A*), *phosphatase and tensin homolog* (*PTEN*) and *Rb*, are often depleted in different types of tumors [4–10]. Therefore, given the direct value of CNVs on gene expression, it is necessary to explore high frequency of CNVs and illuminate the critical genes function in LUAD tumorigenesis.

In this study, we analyzed the specific aberrant CNVs in early-stage LUAD tissues by using whole-genome sequencing and nucleic acid flight mass spectrometry technology, and concentrated to investigate the role of *Cytochrome c oxidase subunit 6C* (*COX6C*), a gene of mitochondrial electron transport chain frequently amplified in LUAD. We found that COX6C was overexpressed in LUAD tissues, and COX6C knockdown (KD) caused mitochondrial dysfunction and ROS accumulation, then activated AMPK signaling pathway and suppressed cell proliferation via modulating mitotic progression.

## Materials

### Patients and LUAD tissues samples

DNA extracted from 38 paired tumor and normal tissues, as well as 10 pneumonia samples were sent for NGS. The tissue microarray (TMA) was prepared by Kunshan Huayi Biotechnology (Kunshan, China), which contained paired tumor tissues and adjacent non-cancerous tissues from 145 cases LUAD patients. In addition, paired tumor and normal tissues from 40 LUAD patients were used to evaluate the expression of COX6C mRNA and protein. All of patients were enrolled from the First Affiliated Hospital of Wenzhou Medical University (Wenzhou, China) with informed consent, as approved by the Research Ethics Committee (2019-ky-50).

### Verification of copy number amplification sequences by MALDI-TOF MS

The copy number of 8q22.1-22.2 and *COX6C* were verified by MALDI-TOF MS as previous reported [11]. SNP sites with high allele frequency were selected for iPLEX assay design (Supplementary Tables S1 and S2). SNP allelotyping was performed by MassARRAY Analyzer 4 with CPM (Agena Biosciences, Shanghai, China) according to the manufacturer’s protocol. The ratio of the peak area of the two alleles in the heterozygous sample (AR) was calculated as follows: AR = area of high molecular weight allele/area of low molecular weight allele. Due to AR for each SNP within two balanced alleles were not always equal to one, we used Z-score to identify CNVs. AR of healthy individuals with normal copy number was used as reference. The mean AR ratio (AR_ref_) and standard deviation of reference (SD_ref_) were calculated, and then the Z-score of each sample with AR_sample_ (normal or tumor tissue) was calculated as follows: Z = (AR_sample_ - AR_ref_)/SD_ref_.

### Immunohistochemistry (IHC)

IHC assays were performed as previously described [12]. Briefly, TMA slide was dewaxed with xylene, and then hydrated by washing with a graded ethanol series and distilled water. The slide was stained with mouse anti-COX6C antibody (1:20 dilution) at 4 °C for 12 h after antigen retrieval and blocking, and then were incubated with HRP-conjugated goat anti-mouse antibodies at room temperature for 30 min. Signal was revealed using 3,3′-diaminobenzidine (3,3′-DAB), and counterstained with hematoxylin. Each tissue was captured using Nikon light microscope (Nikon Eclipse Ci, Tokyo, Japan). The staining intensity of COX6C was scored via IHC Profiler plugin in Image J. The investigator was blinded to the samples during the experiment.

### siRNAs and antibodies

Nonsense control siRNA (siNC: 5′-TTCTCCGAACGTGTCACGTTT-3′) and siRNAs specific targeting to COX6C (si-1: 5′-GCGAAAUCAUAUGGCUGUATT-3′, si-2: 5′-GAAACUACGAUGUCAUGAATT-3′) were synthesized by Genepharma (Shanghai, China).

Mouse anti-COX6C antibody (ab110267), mouse anti-mfn1 antibody (ab126575), mouse anti-mfn2 antibody (ab56889), mouse anti-GRP75 antibody (ab2799), rabbit anti-TOM70 antibody (ab289977), rabbit anti-TOM40 antibody (ab185543) and rabbit anti-TOM20 antibody (ab186735) were purchased from Abcam (Cambridge, UK). Rabbit anti-cyclin B1 antibodies (55004-1-AP) and mouse anti-PARP-1 antibody (66520-1-Ig) were purchased from Proteintech (Rosemont, USA). Mouse anti-α-Tubulin antibody (T5168) and rabbit anti-β-actin antibody (SAB5600204) were purchased from Sigma Aldrich (Billerica, USA). Rabbit anti-cyclin A2 antibody (67955), rabbit anti-cyclin D1 antibody (55506), rabbit anti-CDK2 antibody (18048), rabbit anti-pH3S10 antibodies (9701), rabbit anti-cleaved caspase 7 antibody (8438), rabbit anti-VDAC antibody (4661), rabbit anti-phospho-Aurora A antibody (3079), rabbit anti-phospho-P70 antibody (97596), rabbit anti-P70 antibody (5707), rabbit anti-phospho-AMPK antibody (2535) and rabbit anti-AMPK antibody (5831) were purchased from Cell Signaling Technology (Boston, USA). Mouse anti-Aurora A antibody (610939) and mouse anti-BubR1 antibody (612502) were purchased from BD biosciences (California, USA). Human anti-CREST antibody (HCT-0100) was purchased from ImmunoVision. All secondary antibodies were obtained from Life Technologies Inc. (Massachusetts, USA).

### Cell culture, transfection, and drug treatment

LUAD cells and Beas-2b cells were provided by Dr. Haishang Huang of Wenzhou Medical University (Wenzhou, China) and were characterized by DNA fingerprinting and isozyme detection. Beas-2b, H1299, and H1975 were cultured in RPMI 1640 medium (Gibco, California, USA) supplemented with 10% fetal bovine serum (FBS, Gibco). A549 cells were cultured in Ham’s F12K medium (Gibco) supplemented with 10% FBS. HCC-827 cells were cultured in DMEM medium (Gibco) supplemented with 10% FBS. All cells were cultured at 37 °C in 5% CO_2_. siRNAs and plasmids transfection were conducted with Lipofectamine 3000 (L3000015, Life Technologies Inc.) according to the manufacturer’s protocol.

For Compound C (C.C, HY-13418, MedChemExpress, New Jersey, USA) or Trolox (T1710, Topscience, Shanghai, China) treatment, cells were firstly pretreated with 1 μM C.C for 0.5 h or 400 μM Trolox for 1 h. After siRNA transfection, cells were incubated with medium containing C.C or Trolox for 72 h.

### Construction of stably transfected cell lines

pLKO.1-TRC-copGFP-2A-PURO vector and pLKO.1-TRC-copGFP-2A-PURO-shCOX6C were purchased from Tsingke (Beijing, China). All of the lentivirus vectors were packaged in 293T cells with the psPAX2 and pMD2.G packaging vectors. Lentiviruses were used to infect H1299 cells. Stably transfected cell lines were selected by puromycin for two days. Each shRNA shares the same core sequence with corresponding siRNA.

For establishment the H1299 cells stably expressed Mito-DsRed, pDsRed2-Mito vector was transfected with Lipofectamine 3000, and the cells were selected with neomycin for ten days and cell clones expressing DsRed2 were obtained.

### Immunoblotting, immunofluorescence, and mitotic defects analyses

Cells were harvested or fixed for immunoblotting or immunofluorescence analysis as previously described [12]. In brief, for immunoblotting, cells were lysed in RIPA buffer and equal amounts of total protein lysates were subjected to SDS-PAGE. Immunoblot analysis was performed with corresponding antibodies. For immunofluorescence analysis, cells cultured on glass coverslips were fixed and immunostained with indicated antibodies followed by corresponding secondary antibodies. Images were captured using a Nikon confocal microscope (Nikon A1, Tokyo, Japan).

For mitotic cells counting, at least 2200 cells were analyzed per experiment, and among them, at least 120 mitotic cells were observed and analyzed by their condensed DNA and spindle structure. Mitotic index was calculated as following formula: Mitotic index (%) = number of mitotic cells/number of total cells × 100; the ratio of cell number in each mitotic phase or early mitosis relative to total mitotic cells, the ratio of cells with multipolar spindle in early mitotic cells, the ratio of cells with lagging chromosome in late mitotic cells and the ratio of multinuclear cells in total cells were also calculated.

### RNA isolation and quantitative real-time PCR (qRT-PCR)

RNA isolation and qRT-PCR assays were performed as previously described [12]. One microgram RNA was used for cDNA synthesis and qPCR was performed on a Biorad CFX 96 Touch using SYBR Green (TIANGEN BIOTECH, Beijing, China) as a dsDNA-specific fluorescent dye. β-actin was used for standardizing indicated mRNA level. Amplification primers were synthesized as follow: 5′-GGCGTCTGCGAAATCAT-3′ (forward) and 5′-CAGCCTTCCTCATCTCCTC-3′ (reverse) for *COX6C*, 5′-TGCGTTACACCCTTTCTTGACA-3′ (forward) and 5′- GCAAGGGACTTCCTGTAACAATG-3′ (reverse) for *β-actin*, 5′-GGGGCAGCGGCGTAAGAT-3′ (forward) and 5′-GCTTCTCGGTCTCGGACAAAA-3′ (reverse) for *SOX2*, 5′-GCTCACCCTGGGCGTTCT-3′ (forward) and 5′-CATTGTTGTCGGCTTCCTCC-3′ (reverse) for *OCT4*, 5′-GCTCCGCTCCATAACTTCG-3′ (forward) and 5′-AGGCTTGTGGGGTGCTAAA-3′ (reverse) for *Nanong*. Data were analyzed using the 2^−ΔΔCt^ method.

### Cell proliferation, colony formation, cell cycle analysis, and apoptosis assay

COX6C stably knockdown cells or control cells were seeded on 24-well plates (7000 cell per well), or H1299 or H1975 were seeded on 48-well plates (3000 cell per well) for 24 h and transfected with indicated siRNA. The cells proliferation was determined using cell counting chamber. Colony formation, cell cycle analysis and apoptosis assay were performed as previously described [12].

### Mitochondrial membrane potential (MMP) assay

MMP assay was carried out by using enhanced mitochondrial membrane potential assay kit with JC-1 (Beyotime, Shanghai, China). Cells transfected with indicated siRNA were incubated with JC-1 solution for 20 min at 37 °C. Then, the cells were rinsed twice using JC-1 buffer. Images were captured using a NIKON confocal microscope. The ratio of red to green fluorescence represented the MMP. Fluorescence intensity was measured by Image J.

### Intracellular ROS assay

Detection of intracellular ROS was carried out by using Reactive Oxygen Species Assay Kit (Beyotime). Cells transfected with siRNA were incubated with 10 μM H2DCFDA for 20 min at 37 °C. Then the cells were rinsed twice using serum-free cell culture solution, and applied for flow cytometry using Beckman CytoFLEX (Beckman, California, USA) immediately. Relative fluorescence intensity (RFI) of H2DCFDA was determined as a ratio of the detected fluorescence signals of experimental treatment groups to control groups.

### Determination of mitochondrial ROS

Detection of mitochondrial ROS was carried out by using MitoSox Red Kit (HY-D1055, MedChemExpress). Cells transfected with siRNA were incubated with 10 μM MitoSox Red for 30 min at room temperature. Then the cells were rinsed twice using serum-free cell culture solution, and images were captured using a NIKON confocal microscope immediately. The fluorescent intensity relative to total cells was calculated and RFI was determined as a ratio of the fluorescence signals of experimental treatment groups to control groups.

### Determination of oxygen consumption rate (OCR)

The OCR was determined using an oxygen electrode system Oxygraph-2k (Oroboros, Innsbruck, Austria). The cells were harvested from 6 cm dish and washed with TDS (25 mM Tris-base, 137 mM NaCl, 10 mM KCl, 0.7 mM Na_2_HPO_4_, 10% serum, pH 7.4–7.5) and then centrifugation at 1000 rpm for 5 min. The cells were resuspended in TD (25 mM Tris-base, 137 mM NaCl, 10 mM KCl, 0.7 mM Na_2_HPO_4_, pH 7.4–7.5) and added to the test chamber to record the basal respiratory oxygen consumption. ATP production and maximal oxygen consumption rate were calculated in response to oligomycin (1 μL, 0.1 mg/mL, Sigma-Aldrich, Missouri, USA) and trifluoromethoxy carbonylcyanide phenylhydrazone (FCCP, 1 μL, 0.1 mM, Sigma-Aldrich), respectively. All values were normalized by cell numbers.

### Determination of mitochondrial length

H1299 cells stably expressing DsRed2-Mito were transfected with indicated siRNAs for 72 h. The cells were fixed and stained with DAPI. Images were taken by NIKON confocal microscope. The mitochondrial length in at least 28 cells of each treatment was measured via MiNA-2.0.0 plugin in Image J.

### Transmission electron microscopy

COX6C KD and control H1299 cells were fixed in 2.5% glutaraldehyde (A600875, Sangon Biotech, Shanghai, China) for 2 h at room temperature. After wash, samples were post-fixed with 1% osmium tetroxide (Spi-Chem, West Chester, USA) at 4 °C for 2 h, and dehydrated with a grade series of ethanol. Samples were infiltrated, embedded in Epon-Araldite Resin (Spi-Chem), and polymerized at 60 °C for 48 h. Ultrathin sections of 70 nm were prepared, and then were stained with 3% uranyl acetate and 2.7% lead citrate. Samples were observed with a HT7800 transmission electron microscope (Hitachi, Ltd. Tokyo, Japanese). Mitochondrial length was analyzed by Image J.

### Tumor growth assay in vivo

Animal experiments were approved by the Animal Ethics Committee of Wenzhou Medical University. 5- to 6-weeks-old male BALB/c nude mice were purchased from Vital River Laboratories (Beijing, China). The mice were randomly grouped and three 7 × 10^6^ COX6C stably knockdown cells or control cells were hypodermic injected into the flank of each mouse (each group *n* = 6). Tumors were measured per three days using a vernier caliper, and tumor volume (V) was calculated using the equation V = 1/2ab^2^, where a and b represent the length and width respectively. The investigator was blinded to the group allocation of the animals.

### Statistical analysis

All the statistical analyses were performed by GraphPad Prism version 8.0 (GraphPad Software, California, USA) and SPSS 20.0 statistical software (SPSS Inc., Chicago, USA). Data are presented as the means ± standard deviations from at least 3 independent experiments. Paired student *t*-test was used to assess the significance of COX6C staining in cancers and their coupled adjacent non-cancerous tissues. Chi-square test was performed to evaluate the association between COX6C expression and clinicopathological parameters. Two-sided student *t*-test was used to analyze the difference between two groups. *P* value < 0.05 was considered as statistically significant.

## Results

### Copy numbers of several chromosomal segments are commonly amplified in early-stage LUAD tissues

**Fig. 1:**
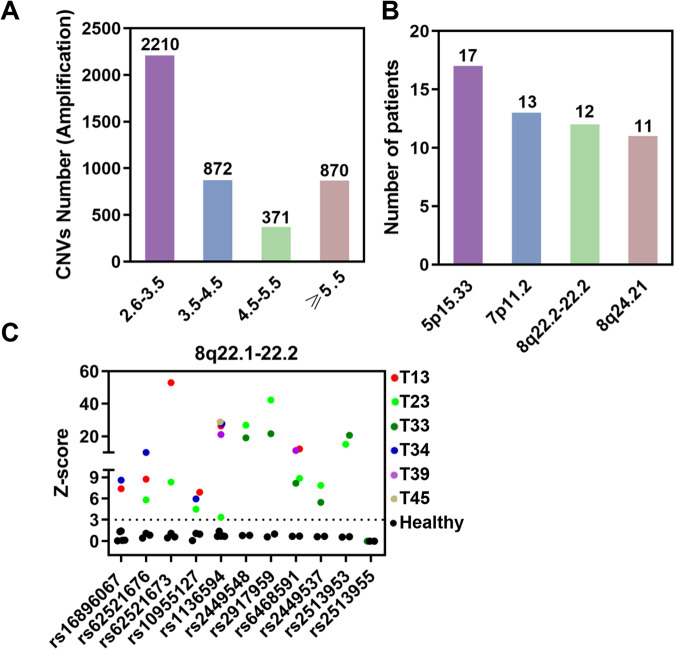
Screening and verification of copy number amplification in lung adenocarcinoma (LUAD). **A**, **B** Low-depth whole-genome sequencing analysis of CNVs in 38 LUAD patients, and bar graph showing the number of regions with different extent increased copy in LUAD tissues (**A**), as well as the number of patients with corresponding CNVs in the 38 LUAD patients (**B**). **C** MALDI-TOF MS to analyze of the CNV of 8q22.1-22.2 in LUAD tissues. Scatterplot showing the absolute values of Z-score for each SNP from 8q22.1-22.2. The plots represent buffy coat DNA from healthy or DNA samples from individual LUAD tissue. The dotted line was the demarcated cut off value.

To elucidate the roles of CNVs in LUAD carcinogenesis, we enrolled 38 cases with early-stage LUAD and 10 cases with pneumonia, and analyzed the abnormalities of copy number in tumor tissues by low-depth whole-genome sequencing. Compared to DNA from pneumonia samples, multiple genomic regions with increased copy number at different extent in LUAD tissues were observed (Fig. 1A). Among them, the copy number of 5p15.33 (Chr 5: 700,021-865,026), 7p11.2 (Chr 7: 54,759,673-55,569,668), 8q22.1-22.2 (Chr 8: 97,728,091-98,103,056) and 8q24.21 (Chr 8: 128,940,154-129,870,067) were separately increased in 17, 13, 12 or 11 of 38 tumor tissues (Fig. 1B). The amplification of 5p15.33, 7p11.2 and 8q24.21 has been reported to be associated with multiple types of cancers [13–16], whereas the role of 8q22.1-22.2 multiplication in cancer was poor investigated. We further validated the amplification of 8q22.1-22.2 using Matrix-assisted laser desorption/ionization time-of-flight mass spectrometry (MALDI-TOF MS), a previous reported method used for CNVs analysis (Fig. 1C) [11]. These results indicate that the abnormalities of gene copy number commonly exist in early-stage LUAD tissues, which are implied to be served as drivers in tumorigenesis.

### *COX6C* is amplified and upregulated in LUAD tissues

**Fig. 2:**
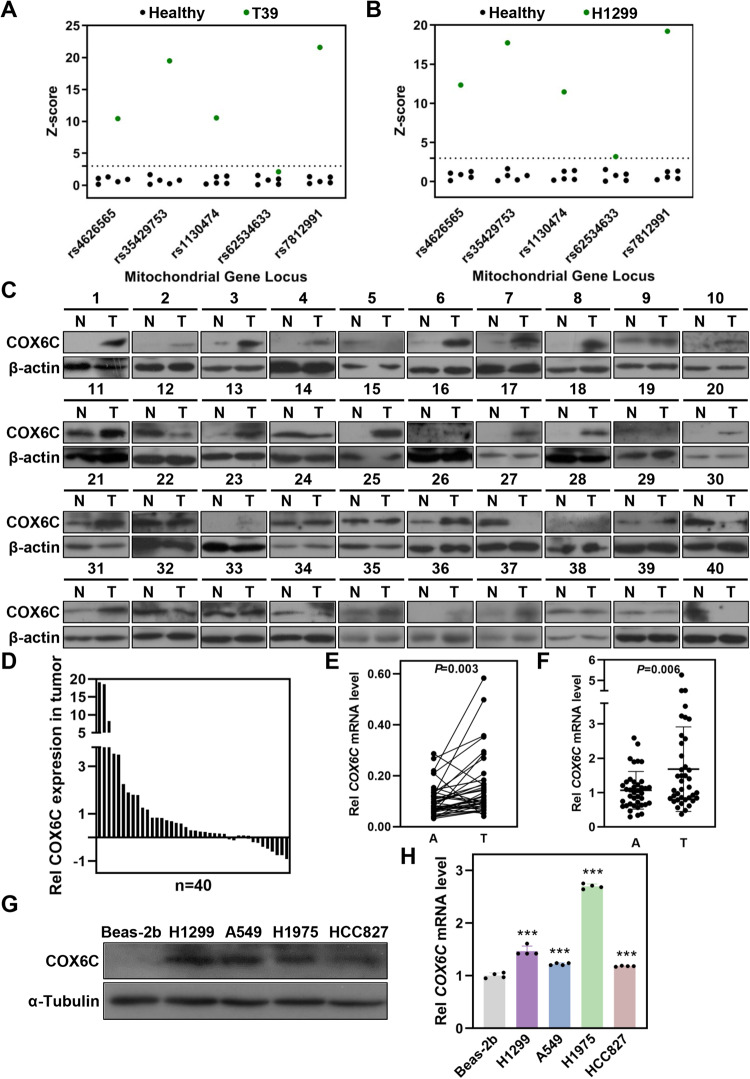
COX6C is amplified and overexpressed in LUAD tissues and cells. **A**, **B** MALDI-TOF MS to analyze the CNV of *COX6C* in LUAD tissue and cells. Scatterplot showing the absolute values of Z-score for individual SNPs of *COX6C*. The black dots represent buffy coat DNA from healthy and the green dots represent DNA from tissue of a LUAD patients (**A**) or H1299 cells (**B**). **C** Immunoblotting showing the expression of COX6C in LUAD tissues (T) and corresponding normal tissues (N). **D** Quantification the density of immunoblotting bands in (**C**), and the relative expression of COX6C to β-actin was calculated. **E**, **F** Scatterplot showing *COX6C* mRNA expression in LUAD (T) and normal tissues (N). Immunoblotting (**G**) or qRT-PCR (**H**) analysis the expression of COX6C in a normal bronchial epithelial cell line (Beas-2b) and indicated LUAD cell lines. Data are represented as mean ± SD. The *P* values were calculated with Student’s *t* test, ***P < 0.001.

Through examining the genes localize on or nearby 8q22.1-22.2 region, we found a gene of mitochondrial electron transport chain*, COX6C* (Chr 8: 99,885,000-99,890,000), is frequently amplified in various cancers by TCGA data, including LUAD (Fig. S1A). We further examined the amplification of *COX6C* in LUAD via MALDI-TOF MS, and confirmed that the copy number of *COX6C* is enhanced in early-stage LUAD tissues and H1299 LUAD cells (Fig. 2A, B). However, we cannot judge the CNVs of other LUAD cells because the SNP locuses in *COX6C* region are homozygous in other LUAD cells (data not shown).

We next analyzed the correlation between CNVs and COX6C expression using TCGA data (http://www.cbioportal.org/), and the data displayed that the expression of *COX6C* is positively correlates with its copy number in LUAD (Fig. S1B). Meanwhile, we investigated the expression of COX6C in LUAD via analyzing the data from UALCAN database (http://ualcan.path.uab.edu/), and the results showed that both protein and mRNA of COX6C are significantly upregulated in LUAD (Fig. S1C, D). We further collected 40 paired LUAD tumor tissues and normal lung tissues, and compared with paired normal tissues, frequently elevated expression of COX6C was detected in tumor tissues by immunoblotting and qRT-PCR assays (Fig. 2C–F). Besides, the similar results were obtained at COX6C protein and mRNA levels in LUAD cells when compared with human bronchial epithelial cell Beas-2b (Fig. 2G, H). Altogether, these results indicate that *COX6C* is commonly amplified and upregulated in LUAD tissues.

### COX6C knockdown inhibits cell proliferation and growth of LUAD cells

**Fig. 3:**
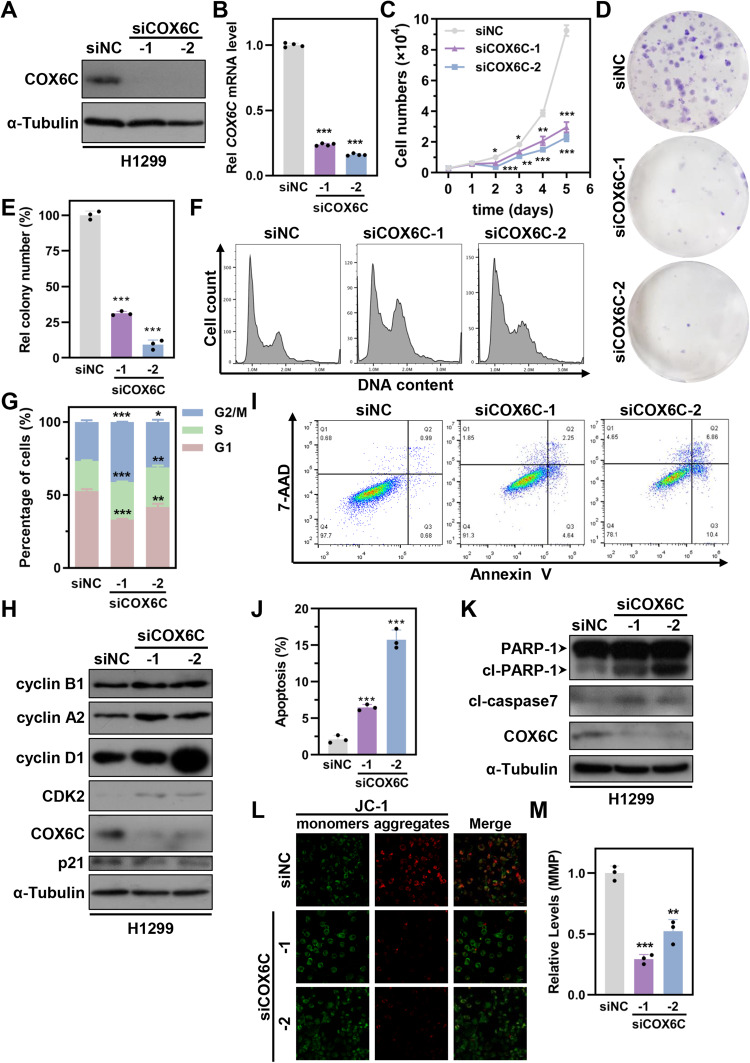
COX6C knockdown (KD) induces cell proliferative inhibition, cell cycle arrest and apoptosis. Immunoblotting (**A**) or qRT-PCR (**B**) analyzed COX6C expression in H1299 cells after transfection with indicated siRNAs for 72 h. **C** Line graph showing the growth curves of H1299 cells after transfected with indicated siRNAs for indicated times. **D** Colony formation analysis of H1299 cells after transfected with indicated siRNAs. **E** The bar graph showing the relative colony number of cells in (**D**). Representative histograms (**F**) and bar graph (**G**) showing the cell cycle distribution of H1299 cells transfected with indicated siRNAs. **H** Immunoblotting showing the expression of cell cycle related proteins in H1299 cells transfected with indicated siRNAs. Representative scatterplot (**I**) and bar graph (**J**) showing the apoptosis of H1299 cells transfected with indicated siRNAs. **K** Immunoblotting showing the expression of apoptotic proteins in H1299 cells transfected with indicated siRNAs. Representative images (**L**) and bar graph (**M**) showing the relative mitochondrial membrane potential (MMP) in H1299 cells transfected with indicated siRNAs. Scale bar: 10 μm. Data are represented as mean ± SD. The *P* values were calculated with Student’s *t* test, *P < 0.05; **P < 0.01 and ***P < 0.001.

To explore the biological function(s) of COX6C in LUAD, COX6C knockdown (KD) were performed by transfection with specific siRNAs, and the results showed that siCOX6C could strikingly inhibit endogenous expression of COX6C in H1299 cells (Fig. 3A, B). Cell count and colony formation assay showed that COX6C KD significantly inhibited the cell proliferative ability of H1299 cells (Fig. 3C–E). Similar results were observed in H1975 cells and confirmed the inhibitory effect of COX6C KD on cell proliferation (Fig. S2A, B).

To investigate the effect of COX6C KD on tumor growth in vivo, stably COX6C KD cell lines were constructed using lentivirus-mediated infection, and the cells displayed an impaired proliferative ability when compared with control cells (Fig. S3A, B). Then the COX6C KD cells and control cells were injected into nude mice, unfortunately, we could not successfully constructed xenograft in nude mice using COX6C KD cells by three times attempt (Fig. S3C). We guessed that COX6C KD may involve in regulating stemness of LUAD cells, and the results showed that COX6C KD effectively inhibited sphere formation and mRNA expression of stemness-related markers including *SOX2*, *OCT4* and *Nanog* (Fig. S3D–F). Collectively, our results identified that COX6C exerts a positive role in regulating tumor proliferation both in vitro and in vivo.

### COX6C suppression mediates cell cycle progression and induces apoptosis in LUAD cells

To further elucidate the approach(es) of COX6C KD-induced cell proliferative suppression, we firstly analyzed the cell cycle distributions. The results showed that COX6C KD induced a strikingly increased accumulation of S-G2/M phase cells and polyploidy cells, while a significant reduction of G1 phase cells in H1299 cells (Fig. 3F, G and S3G). Accordingly, COX6C KD caused a raise of cyclin B1, cyclin A2, cyclin D1 and CDK2 expression (Fig. 3H), which suggested that COX6C KD inhibits cell proliferation via mediating cell cycle progression.

In addition, cell apoptosis was examined in COX6C KD H1299 cells. Annexin V/7-AAD staining results indicated that COX6C KD significantly elevated the proportion of annexin V-positive cells (Fig. 3I, J). Consistently, COX6C KD significantly enhanced the expression of apoptosis-related proteins including cleaved caspase 7 and cleaved PARP-1 (Fig. 3K). COX6C is a subunit of cytochrome c oxidase, which suggests that COX6C KD may cause mitochondrial dysfunction, inducing an abnormality of mitochondrial membrane potential (MMP). To test this possibility, we examined the MMP using JC-1 staining and observed that COX6C KD significantly reduced the level of MMP in H1299 cells (Fig. 3L, M). Therefore, these results showed that COX6C KD causes a reduction of MMP and an increase of cell apoptosis in LUAD cells.

### Knockdown of COX6C induces severe mitotic defects in LUAD cells

**Fig. 4:**
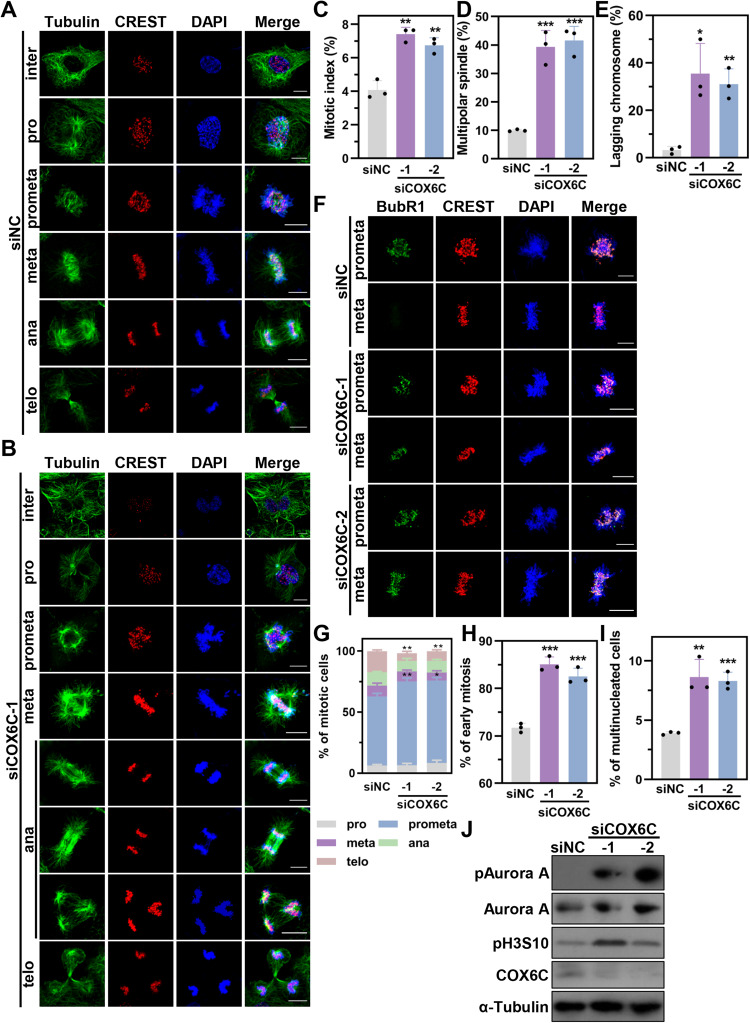
COX6C KD induces mitotic defects in H1299 cells. **A**–**I** H1299 cells were transfected with indicated siRNAs for 72 h, and were fixed for immunostaining. Representative immunostaining images of siNC (**A**) or siCOX6C-1 (**B**) transfected cells at indicated phases. DNA was stained with DAPI. Scale bar: 10 μm. inter: interphase; pro: prophase; prometa: prometaphase; meta: metaphase; ana: anaphase; telo: telophase. Bar graphs showing mitotic index (**C**) and the percentage of cells with multipolar spindle (**D**) or lagging chromosome (**E**) after COX6C KD. **F** Representative images of spindle assembly checkpoint activation after COX6C KD by using immunostaining with BubR1 antibody. DNA was stained with DAPI. Scale bar: 10 μm. Bar graphs showing the percentage of cells in each mitotic phase (**G**) or early mitotic cells (**H**) in mitotic cells, as well as multinucleated cells in all cells (**I**). **J** Immunoblotting showing the expression of mitotic related proteins in H1299 cells after COX6C KD. Data are represented as mean ± SD. The *P* values were calculated with Student’s *t* test, *P < 0.05; **P < 0.01 and ***P < 0.001.

As previous mentioned, COX6C KD resulted in an apparent increase of polyploidy cells (Fig. 3F, G and S3G). Meanwhile, we also observed more than 7% rounded cells when COX6C depleted (Fig. S4A, B). Therefore, we speculated that COX6C KD might induce a mitotic abnormity and led to cell cycle arrest. To test the speculation, immunostaining was performed to assess the mitotic progression of COX6C KD cells (Fig. 4A, B and S4C). The results showed that mitotic index was significantly increased from 4.08 ± 0.46% in siNC cells to 7.40 ± 0.34% in siCOX6C-1 cells or 6.74 ± 0.37% in siCOX6C-2 cells (Fig. 4C and Table 1). The mitotic delay was associated with an enhancement of multipolar spindle, which accompanied with abnormal chromosome congression in prometaphase, nonaligned chromosomes in metaphase, as well as chromosome bridge and lagging chromosomes in anaphase (Fig. 4A–E, S4C and Table 1). These severe mitotic defects inevitably led to activation of the spindle-assembly checkpoint (SAC) in COX6C depleted metaphase cells, as judgment by BubR1, a known component of SAC (Fig. 4F). Consistently, COX6C KD induced an accumulation of prometaphase cells and multinucleated cells, but a decrease in telophase cells (Fig. 4G–I and Table 1). Aurora kinases have been known to play critical roles in mitosis via regulating centrosomal maturation, spindle assembly, chromosome congression and segregation [17]. We found that COX6C KD significantly elevated the levels of phospho-Aurora A and the phosphorylation of H3 at Serine 10 (pH3S10), which is the preferred substrate for Aurora B (Fig. 4J) [18]. The phenotypes including increased round cells and mitotic defects were also observed in H1975 cells after COX6C KD (Fig. S5 and Table S3). Altogether, these results intensively indicate that COX6C KD induces mitotic defects in spindle assembly and chromosome congression, suppressing mitotic progression.

**Table 1:** Summary of the effects COX6C knockdown on mitosis in LUAD cells.

Parameter/Treatment	siNC	siCOX6C-1	siCOX6C-2
Mitotic index (%)	4.08 ± 0.46	7.40 ± 0.34 (**)	6.74 ± 0.37 (**)
Prometaphase (%)	56.66 ± 2.38	68.73 ± 2.66 (**)	67.63 ± 2.43 (*)
Telophase (%)	17.21 ± 1.14	6.39 ± 1.42 (**)	7.98 ± 1.16 (**)
Early mitosis (%)	71.71 ± 0.77	85.10 ± 1.25 (***)	82.54 ± 1.40 (***)
Multipolar spindle in Early mitosis cells (%)	9.94 ± 0.29	39.29 ± 4.72 (***)	41.59 ± 4.05 (***)
Lagging chromosome in late mitosis cells (%)	3.22 ± 1.25	35.47 ± 10.38 (*)	31.05 ± 5.31 (*)
Multinuclear cell (%)	3.90 ± 0.10	8.63 ± 1.22 (**)	8.29 ± 0.60 (***)

The *P* values were calculated with Student’s t test, *P < 0.05; **P < 0.01 and ***P < 0.001 compare to siNC.

### Depletion of COX6C elicits mitochondrial dysfunction and ROS accumulation

**Fig. 5:**
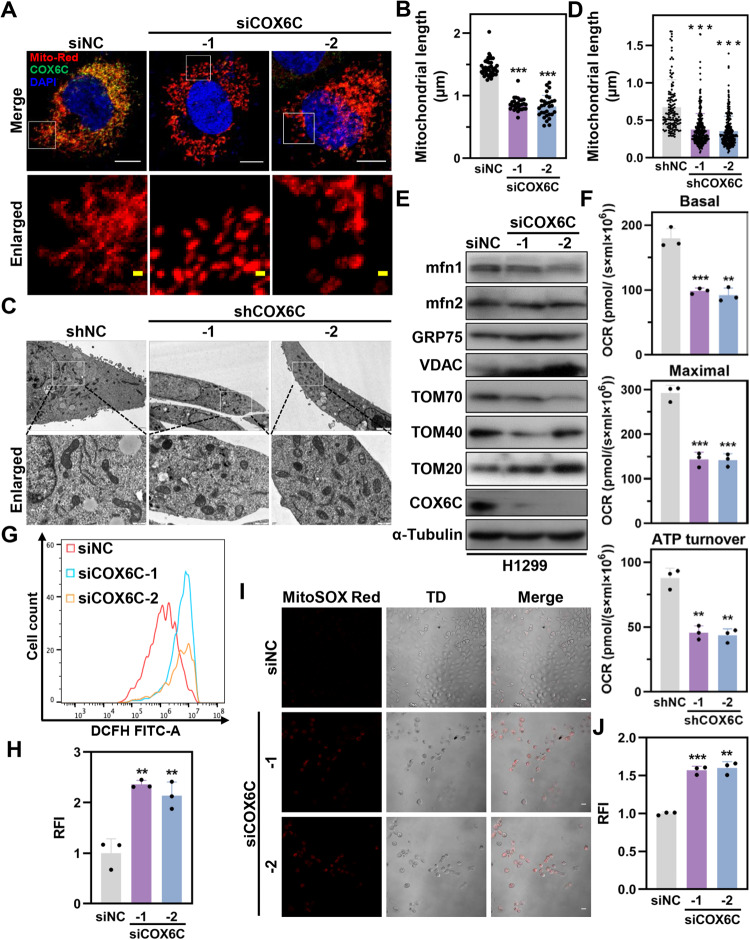
COX6C KD triggers mitochondrial dysfunction and ROS accumulation. **A** Representative images of mitochondrial phenotype in COX6C KD and control cells. Scale bars: 10 µm in the top panel and 1 µm in the bottom panel. **B** Bar graph showing mitochondrial length of H1299 cells in (**A**) (*n* ≥ 28). **C** Representive images showing mitochondrial morphology in H1299 KD and control cells by TEM. Scale bars: 2 µm in the top panel and 500 nm in the bottom panel. **D** Scatterplots showing the mitochondrial length of H1299 cells in (**C**) (*n* ≥ 28). **E** Immunoblotting showing the effects of COX6C KD on expression of mitochondrial proteins. **F** Bar graphs showing the effects of COX6C KD on basal respiration, maximal respiration and ATP turnover. **G** Representative histograms showing the effects of COX6C KD on total cellular ROS production. **H** Bar graph showing the relative fluorescent intensity (RFI) of H2DCFDA in (**G**). **I** Representative images showing the effects of COX6C KD on mitochondrial ROS production by MitoSOX assay. TD transmission detector. Scale bar: 10 μm. **J** Bar graph showing the RFI of MitoSOX Red in **I**. Data are represented as mean ± SD. The *P* values were calculated with Student’s *t* test, **P < 0.01 and ***P < 0.001.

Given that COX6C is a subunit of cytochrome c oxidase and plays a critical role in the mitochondrial electron transport chain, it was mandatory to evaluate whether COX6C KD led to aberrance in mitochondrial metabolic functions. To evaluate whether COX6C KD causes a change in mitochondria structure, we established a H1299 cell line with stably expressed Mito-DsRed2, a discosoma red fluorescent protein fused to a mitochondrial targeting sequence [19]. Surprisedly, compared with control cells, COX6C KD triggered severe mitochondrial fragmentation in the cells, resulting in small and punctuate mitochondria and mitochondrial length decreased from 1.45 ± 0.14 μm in siNC cells to 0.86 ± 0.10 μm in siCOX6C-1 cells or 0.83 ± 0.17 μm in siCOX6C-2 cells (Fig. 5A, B). Similarly, COX6C KD also induced mitochondrial fragmentation in H1975 cells (Fig. S6A, B). We further examined the mitochondrial morphology using transmission electron microscopy (TEM), and the results displayed that, compared with controls, shorter mitochondria were more frequently observed in COX6C KD cells (Fig. 5C, D). Consistently, the expression of pro-fusion GTPase mfn1 was significantly decreased after COX6C KD in both H1299 and H1975 cells (Fig. 5E and S6C).

OXPHOS is one of the most important function of mitochondria and critical for cell proliferation. Therefore, we sought to explore the effects of COX6C KD on energy metabolism via measuring oxygen consumption rate (OCR), an important indicator of OXPHOS, by using Oxygraph-2k. The results showed that COX6C KD resulted in a significant decrease of OCR in basal respiration, maximal respiration and ATP turnover in H1299 cells (Fig. 5F). Besides, COX6C KD led to a significantly increase of GRP75, VDAC-1, and TOM20, but a striking decrease of TOM70 and Tom40 (Fig. 5E). Given the crucial role of mitochondria in cellular redox homeostasis, COX6C-mediated mitochondrial dysfunction also triggered a significant increase of both total cellular ROS and mitochondrial ROS production (Fig. 5G–J and S6D–G). Collectively, these results suggest that COX6C KD induces aberrancy in mitochondrial dynamics and metabolism, thereby promotes ROS accumulation and cell proliferation inhibition.

### AMPK activation is responsible for COX6C-mediated cell responses

**Fig. 6:**
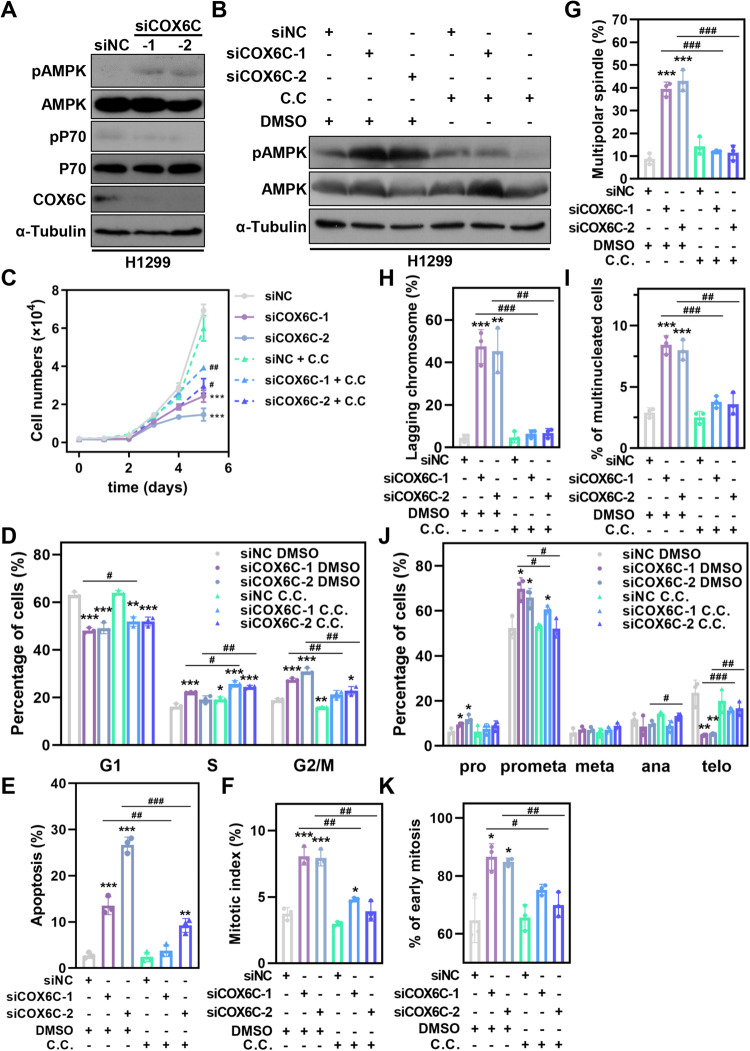
AMPK activation is responsible for COX6C-mediated cell proliferation inhibition and mitotic defects. **A** Immunoblotting showing AMPK activation after COX6C KD. **B**–**K** COX6C KD or control H1299 cells were treated with 1 μM C.C, immunoblotting detecting AMPK activation (**B**), Line graph showing the growth curves (**C**), Bar graphs showing the cell cycle distribution (**D**), percentage of apoptotic cells (**E**), mitotic index (**F**), the percentage of cells with multipolar spindle (**G**) or lagging chromosome (**H**), the percentage of mitotic cells in all cells (**I**), early mitotic cells in mitotic cells (**J**) and multinucleated cells in all cells (**K**). Data are represented as mean ± SD. The *P* values were calculated with Student’s *t* test, *P < 0.05; **P < 0.01 and ***P < 0.001.

Theoretically, the decrease of ATP levels in COX6C KD cells would lead to an elevated amount of adenosine monophosphate (AMP) in cells, which has been well recognized as a prerequisite for activation of AMP-activated protein kinase (AMPK) [20]. To confirm this, immunoblotting was performed, and the results displayed that the phosphorylation of AMPKα1 at Threonine 172 was markedly increased when COX6C was depleted, while the phosphorylation of p70, a substrate of mTOR, was significantly decreased (Fig. 6A and S2A).

To explore whether activated AMPK serves as a downstream effector to modulate COX6C-mediated cell phenotypes, COX6C KD and control cells were cultured in presence or absence of AMPK inhibitor dorsomorphin (Compound C, C.C). The results showed that C.C treatment significantly reduced the induction of AMPK signaling in COX6C KD cells (Fig. 6B). As expected, C.C treatment effectively blocked the proliferative suppression and cell cycle arrest, and rescued cell apoptosis induced by COX6C KD in H1299 cells (Fig. 6C–E and S7A–D).

AMPK was also reported to modulate mitotic progression via phosphorylating several substrates including Kif4A and MRLC (myosin regulatory light chain) [21, 22]. Therefore, we subsequently examined the role of AMPK activation in COX6C-mediated mitotic defects. The immunofluorescence results showed that, after C.C treatment, mitotic defects induced by COX6C KD including mitotic delay, spindle abnormality, and chromosome segregation defects etc. could be significantly reversed (Fig. 6F–K, S7E and Table 2), implying that activated AMPK signaling also is required for COX6C KD induced mitotic defects. These results indicate that AMPK activation plays a vital role in COX6C KD-mediated cell proliferation inhibition, cell cycle arrest, cell apoptosis and mitotic defects in H1299 cells.

**Table 2:** The effects of C.C. treatment on COX6C-mediated mitotic deficiency.

Parameter/Treatment	DMSO			C.C.		
	siNC	siCOX6C-1	siCOX6C-2	siNC	siCOX6C-1	siCOX6C-2
Mitotic index (%)	3.74 ± 0.37	8.07 ± 0.55 (***)	7.94 ± 0.49 (***)	2.97 ± 0.15	4.81 ± 0.13 (*; ##)	3.92 ± 0.60 (##)
Prometaphase (%)	52.37 ± 3.89	69.88 ± 3.98 (*)	65.88 ± 3.37 (*)	53.20 ± 0.87	60.68 ± 1.40 (*; #)	52.08 ± 3.42 (#)
Telophase (%)	23.58 ± 4.59	4.82 ± 0.22 (**)	5.33 ± 0.18 (**)	20.00 ± 4.12	15.85 ± 0.91 (###)	16.70 ± 2.48 (##)
Early mitosis (%)	64.68 ± 6.30	86.61 ± 3.73 (*)	84.88 ± 1.11 (*)	65.71 ± 3.62	75.28 ± 1.48 (#)	70.03 ± 3.48 (##)
Multipolar spindle in Early mitosis cells (%)	8.85 ± 1.96	39.51 ± 2.54 (***)	43.05 ± 3.76 (***)	14.37 ± 3.17	12.10 ± 0.36 (###)	11.52 ± 2.68 (###)
Lagging chromosome in late mitosis cells (%)	4.49 ± 1.41	47.44 ± 6.54 (***)	45.33 ± 8.58 (**)	4.77 ± 2.18	6.48 ± 1.51 (###)	6.79 ± 1.75 (##)
Multinuclear cell (%)	2.91 ± 0.31	8.43 ± 0.61 (***)	8.00 ± 0.68 (***)	2.53 ± 0.39	3.78 ± 0.40 (###)	3.61 ± 0.73 (##)

The *P* values were calculated with Student’s t test, *P < 0.05; **P < 0.01 and ***P < 0.001 compare to siNC. ^#^*P* < 0.05; ^##^*P* < 0.01 and ^###^*P* < 0.001 compare to corresponding siRNA with DMSO treatment.

### COX6C-mediated AMPK activation partially depends on mitochondrial dysfunction-induced ROS accumulation

**Fig. 7:**
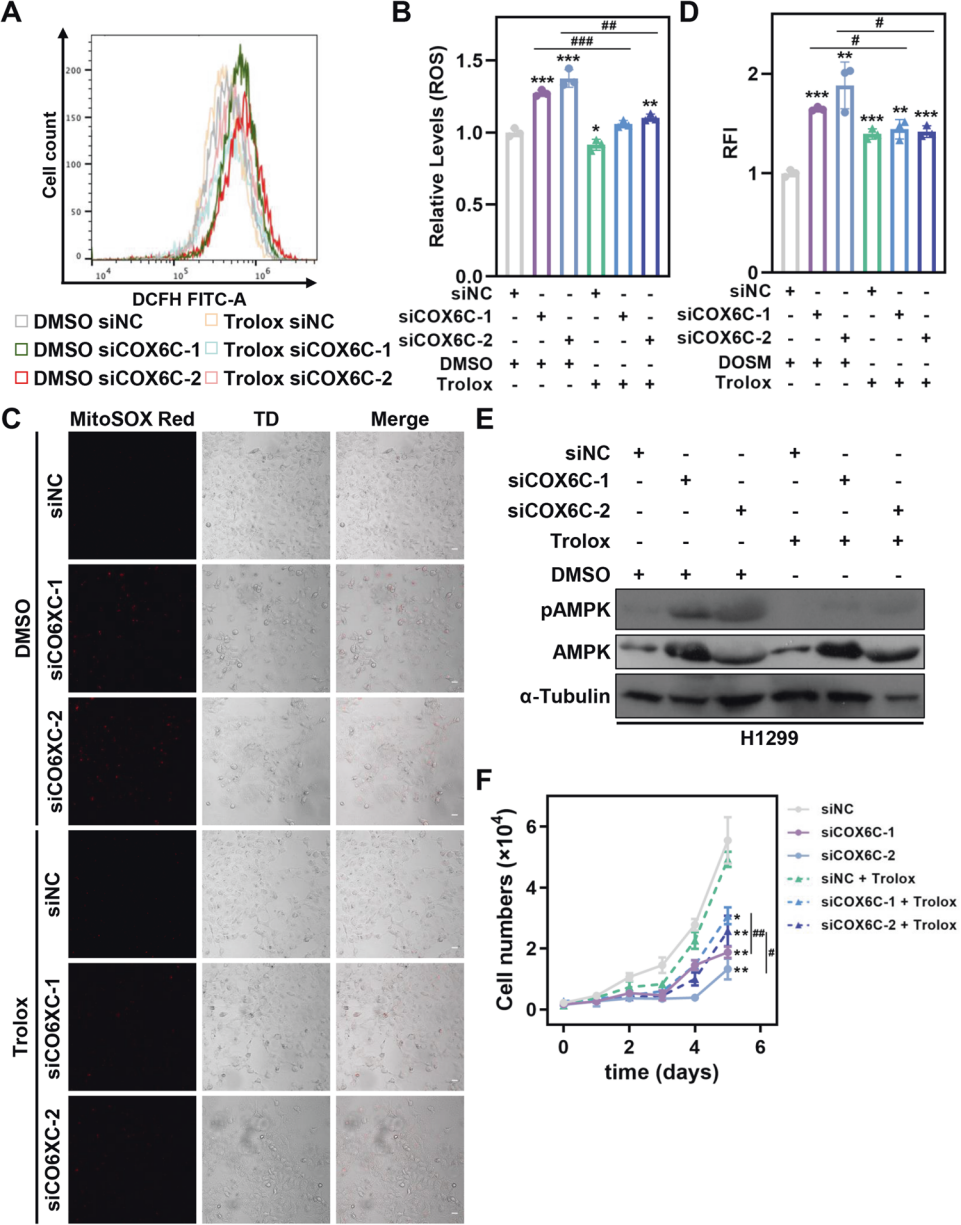
COX6C KD-induced ROS accumulation facilitates AMPK activation. **A**–**F** COX6C KD and control H1299 cells were treated with 400 μM Trolox. Representative histograms (**A**) and bar graph (**B**) showing the level of ROS in the cells. Representative images (**C**) and bar graph (**D**) showing the effects of Trolox treatment on COX6C-mediated mitochondrial ROS production. Scale bar: 10 μm. **E** Immunoblotting showing the effects of Trolox treatment on COX6C KD-induced AMPK activation. **F** Line graph showing the effects of Trolox treatment on COX6C KD-induced cell proliferative inhibition. Data are represented as mean ± SD. The *P* values were calculated with Student’s *t* test, *P < 0.05; **P < 0.01 and ***P < 0.001.

It is well known that ROS accumulation could activate AMPK signaling pathway to regulate multiple cellular response [23]. Thus, we sought to investigate whether mitochondrial dysfunction-induced ROS accumulation after COX6C KD contributes to AMPK activation. Trolox, a ROS scavenger, significantly reduced the ROS accumulation induced by COX6C KD in H1299 cells (Fig. 7A–D). Meanwhile, the AMPK activation and cell proliferative inhibition induced by COX6C KD were partially but significantly reversed by Trolox treatment (Fig. 7E, F). In general, we clarify that COX6C KD-mediated AMPK activation is partially depend on mitochondrial dysfunction-induced ROS accumulation.

### COX6C expression is positively correlated with TNM stage and worse prognosis of patients with LUAD

**Fig. 8:**
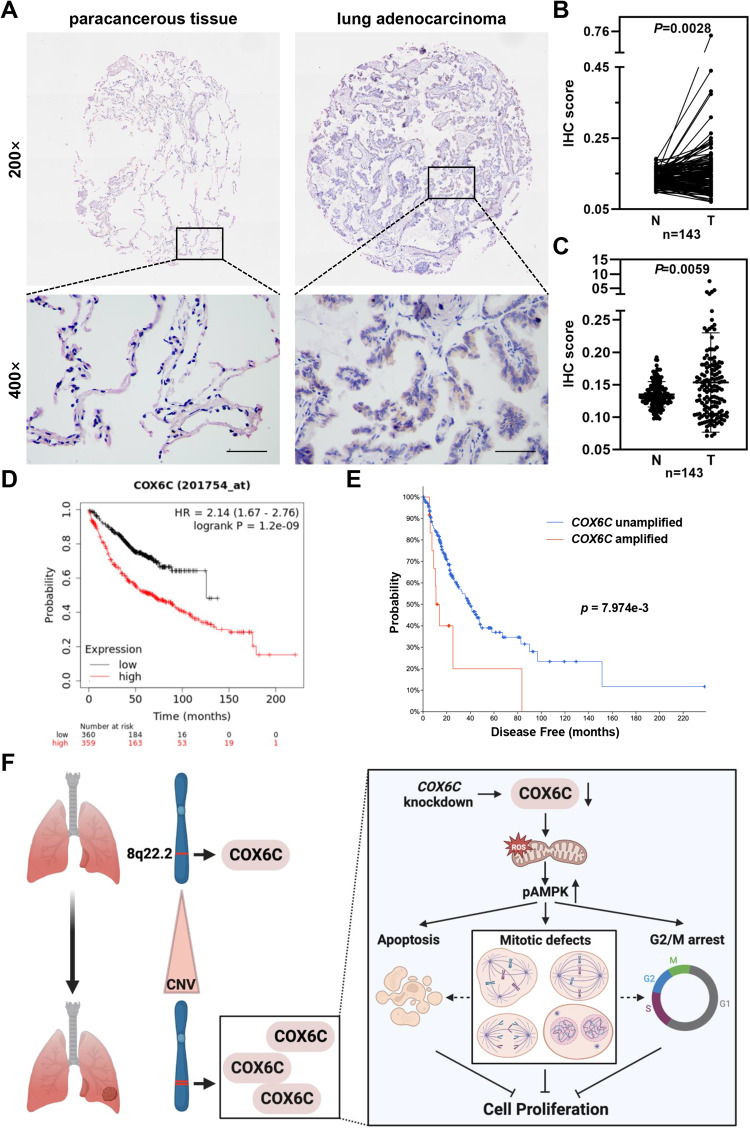
Higher expression of COX6C positively correlates with worse prognosis of LUAD patients. **A** Representative immunohistochemically staining images of COX6C in lung adenocarcinoma and paracancerous tissues of LUAD patients. Scale bar: 100 μm. **B**, **C** The COX6C staining intensity was quantified by calculating IOD, and scatterplot showing the relative expression of COX6C in tumor (T) and normal tissues (N). **D** Survivorship curve showing the relationship between COX6C expression and overall survival (OS) of LUAD patients in TCGA cohort. **E** Survivorship curve showing the relationship between *COX6C* amplification and disease-free survival (DFS) of LUAD patients in TCGA cohort. **F** The model of copy number amplification mediated-COX6C regulates cell proliferation in LUAD. Log-rank test for OS analysis, student’s *t* test for others.

Given the critical role of COX6C in regulating mitochondrial functions and cell proliferation, its abnormal expression probably closely correlates with clinical cancer progression. Therefore, we further evaluated the relationship between COX6C expression and clinicopathological features of LUAD patients using a tissue cohort. Consistent with previous results, COX6C is upregulated in most tumor tissues of LUAD patients (85/143, 59.44%) (Fig. 8A–C). Results of statistical analysis showed that the expression of COX6C is positively correlated with T stage and TNM stage, but not with gender, age and N stage (Table 3). Moreover, higher COX6C expression predicts worse overall survival (OS) of LUAD patients (Fig. 8D), and the role of COX6C in prognostic prediction was further supported by worse disease-free survival (DFS) of LUAD patients with *COX6C* amplification (Fig. 8E). These results indicate COX6C might be a malignant biomarker used for diagnosis and prognostic prediction in LUAD.

**Table 3:** The relationship between COX6C expression and clinicopathological features of lung adenocarcinoma patients.

Variables	COX6C expression		χ^2^	*P* value
	Low	High		
Gender			1.077	0.299
Female	77	14		
Male	42	12		
Age (years)			0.684	0.408
<64	58	15		
≥64	61	11		
T stage			16.213	0.000
T1-T2	109	16		
T3-T4	10	10		
N stage (*n* = 144)			0.901	0.342
N0	92	18		
N1-N2	26	8		
TNM stage			4.473	0.034
I	85	13		
II-IV	34	13		

## Discussion

In this study, we analyzed the CNVs in patients with early-stage LUAD, and explored the cellular functions of a gene, *COX6C*, that localizes on a recurrent amplified region. We demonstrated that frequently amplified copy number and elevated expression of *COX6C* in a high proportion of LUAD. COX6C is essential for mitochondrial function and COX6C KD induces mitochondrial dysfunction, impaired OXPHOS and ROS accumulation, which promotes AMPK activation, inducing mitotic defects and cell proliferative inhibition (Fig. 8F).

It is well implicated that CNVs-mediated gene expression aberrations contribute to cancer progression [24, 25]. For example, *CCND1*, a known oncogene localizes on 11q13, is usually amplified in several types of cancer, such as breast cancer, bladder cancer, and esophageal squamous cell carcinoma [26–28]. *PTEN*, a tumor suppressor gene localizes on 16q23, is frequently deleted in prostate cancer, which is known as an independent prognostic marker of patients with prostate cancer [29]. Here we discovered that several gene regions including 5p15.33, 7p11.2, 8q24.21, and 8q22.1-22.2 are frequently amplified in early-stage LUAD tissues, implies that these regions may play important roles in LUAD tumorigenesis. Among them, 7p11.2 and 8q24.21 have been reported to amplify in non-small cell lung carcinoma (NSCLC), and genes like *EGFR* at 7p11.2 and *myc* at 8q24.21 were widely regarded as driver genes of NSCLC [30, 31]. In addition, *telomerase reverse transcriptase* (*TERT*), a gene localizes on 5p15.33, has been found to amplify in 4.6% of anaplastic lymphoma kinase positive (ALK+) lung cancer patients [32]. Here, we explored the oncogenic activity of *COX6C*, a gene localizes on a newfound amplicon, 8q22.2, in LUAD.

COX6C is one of the most important components of the terminal enzyme of the respiratory chain, which is coded by nuclear genome and transported to inner mitochondrial membrane. It has been well known that COX6C plays a pivotal role in OXPHOS, and several evidences suggest that COX6C is closely correlated with various diseases. For example, COX6C is downregulated in blood samples from familial hypercholesterolemia patients as well as temporal and parietal cortices in patients with Alzheimer’s disease, while is significantly elevated in the glomeruli of diabetic nephropathy rats [33–36]. In addition, increased expression of COX6C was also observed in cancerous tissues from patients with prostate cancer or estrogen receptor positive (ER+) breast cancer [37, 38]. However, the mechanism of COX6C upregulation in cancers and its role in tumorigenesis remain elusive. Here, we found that *COX6C* is frequently amplified and its expression is positively correlated with the copy number and elevated in LUAD. COX6C promotes LUAD carcinogenesis via regulating mitochondrial function and cell cycle progression. In addition, We found COX6C is frequently overexpressed in most of tumor tissues from LUAD patients, but the amplification of *COX6C* is only observed in about 4.1% LUAD patients according to TCGA data. Hence, we could not eliminate that there are other possibilities contribute to increased expression of COX6C in LUAD carcinogenesis. Indeed, COX6C has been reported to be regulated by Estrogen-Related Receptor α (ERRα), NRF-1/2 or miR-4276 at transcriptional or post-transcriptional level [39–43]. ERR-α and NRF-1/2 have been demonstrated to participate in lung adenocarcinoma progression [44]. The role of COX6C in these factors-mediated tumorigenesis remains need to further investigation.

Mitochondria, central intracellular hubs of metabolism and redox maintenance, play a vital role in balancing oxidative stress-induced cell death and cell proliferation in cancer cells. Abnormalities in mitochondrial structure, function and dynamics would induce multiple serious consequence, such as excessed ROS accumulation [45]. ROS could irreversibly induce damage of proteins, lipids and DNA [46]. It is also well established that multiple signaling pathways, including MAPK, AKT, and AMPK, etc. could be activated responding to excessed ROS, then regulates diverse cell behaviors, such as cell cycle arrest and cell death [23, 47]. Here, we found that COX6C KD induced abnormity in mitochondrial structure and OXPHOS impairment, inducing mitotic defects and cell proliferative inhibition via activating AMPK pathway. However, ROS scavenger cannot completely reverse COX6C-mediated cell responses and AMPK activation, indicates that there are other factors may contributes to COX6C KD-induced AMPK activation.

In conclusion, we found that COX6C, a frequently amplified gene localizes to 8q22.1-22.2, is upregulated in tumor tissues and positively correlates with poorer prognosis of LUAD patients. COX6C is essential for mitochondrial function, and COX6C KD promotes ROS accumulation and activates AMPK signaling, suppressing cell proliferation via inducing mitotic defects, G2/M arrest and apoptosis, which serves COX6C as an attractive biomarker used for prognostic prediction and therapeutic target in LUAD patients.

### Reporting summary

Further information on research design is available in the Nature Research Reporting Summary linked to this article.

### Supplementary information

Reporting Summary

Supplemental data

Original Data File

## Data Availability

The datasets generated in the current study are available from the corresponding authors on reasonable request.
