# Supplementary material for: COX6C expression driven by copy amplification of 8q22.2 regulates cell proliferation via mediation of mitosis by ROS-AMPK signaling in lung adenocarcinoma
Source: Cell Death Dis. 2024 Jan 19;15(1):74. doi: 10.1038/s41419-024-06443-w (PMC10799076; doi:10.1038/s41419-024-06443-w)
Supplement: Supplementary file 2 — Supplemental data [file 41419_2024_6443_MOESM2_ESM.docx]

**Fig. S1 *COX6C* is a frequently amplified gene and high expressed in LUAD.** (A) Bar graph showing the alteration of *COX6C* in different types of cancer using TCGA data analysis. (B) Scatterplots showing the relationship between the expression of COX6C mRNA and its copy number. Bar graphs showing that the expression of COX6C protein (C) or mRNA (D) in LUAD tissues and normal tissues using indicated online data. The *P* values were calculated with Student’s *t* test, **P*< 0.05; ***P*< 0.01 and ****P*< 0.001.

**Fig. S2 COX6C positively regulates cell proliferation of LUAD cells.** (A) Immunoblotting showing the expression of indicated proteins in H1975 KD cells and control cells. (B) Line graph showing the growth curves of H1975 cells after transfected with indicated siRNAs. Data are represented as mean ± SD. The *P* values were calculated with Student’s *t* test, **P*< 0.05; ***P*< 0.01 and ****P*< 0.00.

**Fig. S3 COX6C KD inhibits cell proliferation and impairs the stemness of H1299 cells.** (A) Immunoblotting showing the expression of COX6C in H1299 cells with stably COX6C KD or controls. (B) Line graph showing the growth curves of H1299 cells with stably COX6C KD or controls. (C) Image of subcutaneous xenograft tumors formed by control H1299 cells in nude mice (n=6). Representative images (D) and bar graph (E) showing the inhibition of COX6C KD on colony formation using soft agar assay. Scale bar: 50 μm. (F) qRT-PCR analysis the effect of COX6C KD on the expression of stemness-related genes. (G) Bar graph showing the percentage of polyploid cells after COX6C KD. Data are represented as mean ± SD. The *P* values were calculated with Student’s *t* test, **P*< 0.05; ***P*< 0.01 and ****P*< 0.001.

**
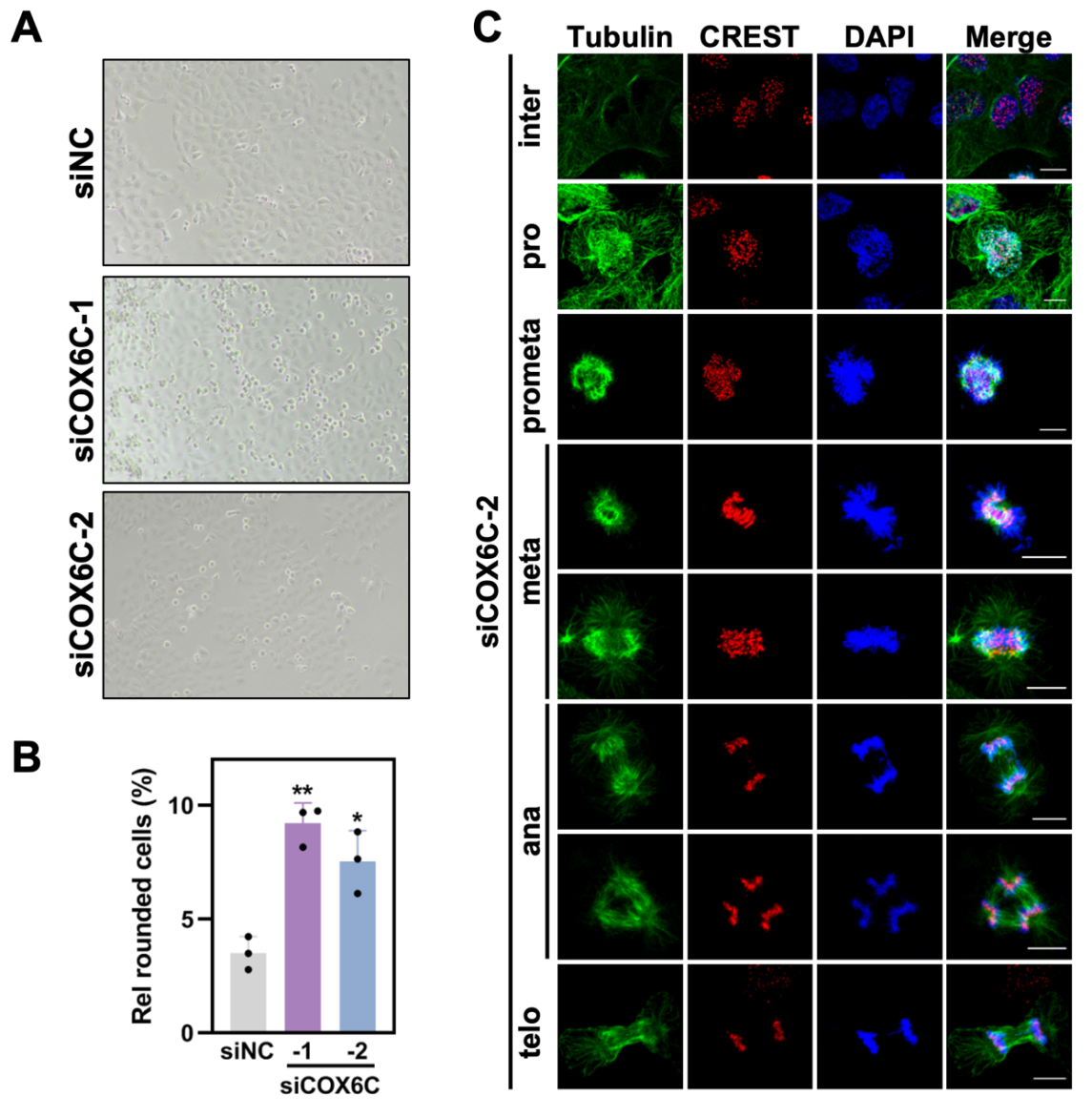
**

**Fig. S4 COX6C KD induces mitotic defects in H1299 cells.** (A-C) H1299 cells were transfected with indicated siRNAs for 72 h. (A) Representative images of cultured COX6C KD and control cells under optical microscope. (B) Bar graph showing the percentage of rounded cells from A. (C) Representative immunostaining images of siCOX6C-2 transfected cells at indicated phases. Scale bar: 10 μm. Data are represented as mean ± SD. The *P* values were calculated with Student’s *t* test, **P*< 0.05 and ***P*< 0.01.

**Fig. S5 COX6C KD induces mitotic defects in H1975 cells.** (A-C) H1975 cells were transfected with indicated siRNAs for 72 h. (A) Representative images of cultured COX6C KD H1975 and control cells under optical microscope. (B) Bar graph showing the percentage of rounded cells from A. (C-E) Representative immunostaining images of H1975 cells transfected with indicated siRNAs at different phases. Scale bar: 10 μm. inter: interphase; pro: prophase; prometa: prometaphase; meta: metaphase; ana: anaphase; telo: telophase. Bar graphs showing mitotic index (F) and the percentage of cells with multipolar spindle (G) or lagging chromosome (H), cells in each mitotic phase (I) or early mitotic cells (J) in mitotic cells, as well as multinucleated cells in all cells (K) after COX6C KD. Data are represented as mean ± SD. The *P* values were calculated with Student’s *t* test, **P*< 0.05 and ***P*< 0.01.

**Fig. S6 COX6C KD triggers mitochondrial dysfunction and ROS accumulation in H1975 cells.** (A) Representative images of mitochondrial phenotype in COX6C KD and control H1975 cells. Scale bar: 10 µm in the left panel and 1 µm in the right panel. (B) Bar graph showing mitochondrial length of H1975 cells in A (n≥18). (C) Immunoblotting showing the effects of COX6C KD on indicated mitochondrial pro-fusion GTPases. (D) Representative histograms showing the effects of COX6C KD on total cellular ROS production in H1975 cells. (E) Bar graph showing the relative fluorescent intensity (RFI) of H2DCFDA in D. (F) Representative images showing the effects of COX6C KD on mitochondrial ROS production by MitoSOX assay. TD: Transmission detector. Scale bar: 10 μm. (G) Bar graph showing the RFI of MitoSOX Red in F. Data are represented as mean ± SD. The *P* values were calculated with Student’s *t* test, ***P*< 0.01 and ****P*< 0.001.

**
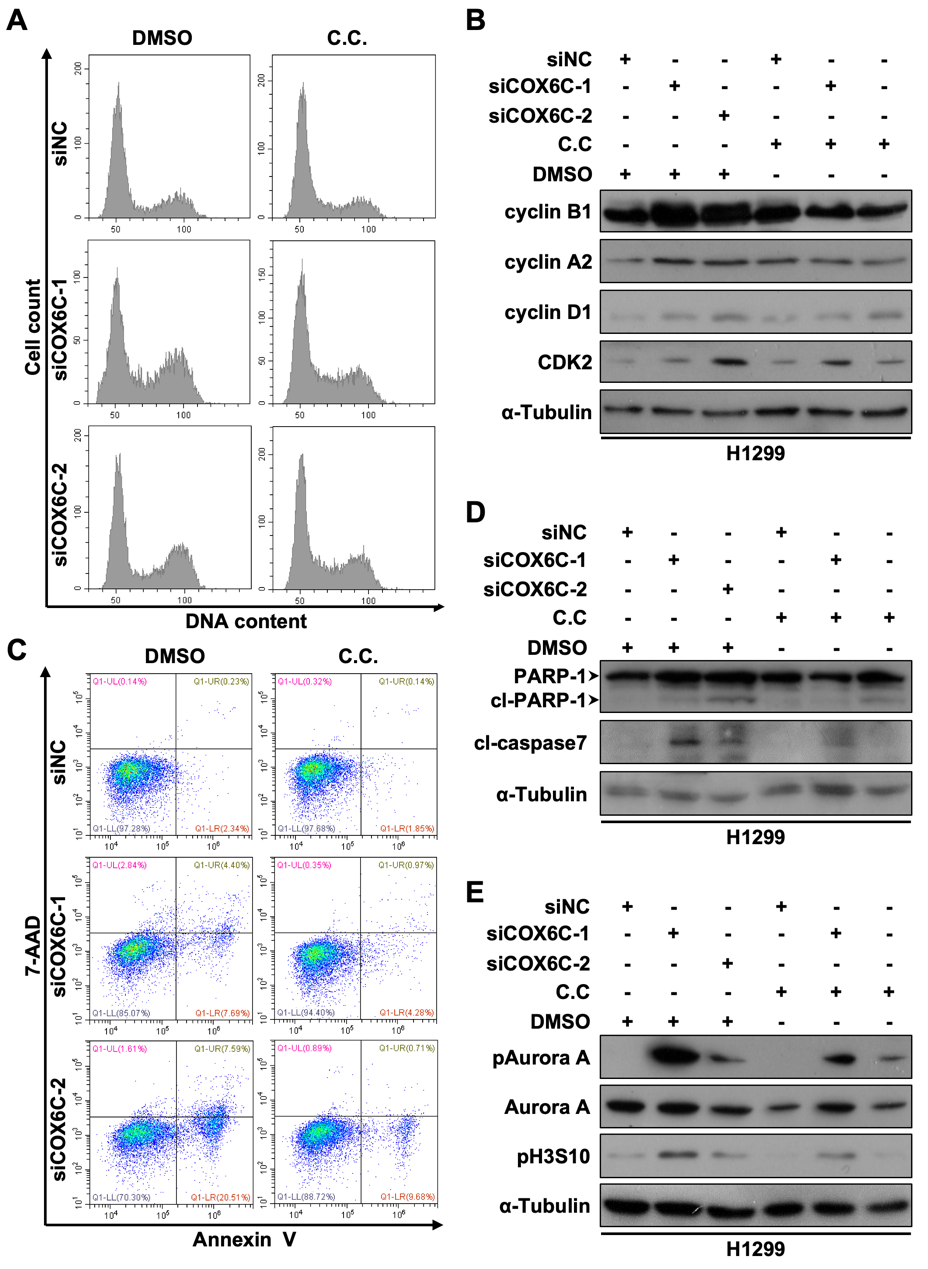
**

**Fig. S7 AMPK activation is essential for COX6C-mediated cell cycle arrest and apoptosis.** COX6C KD or control H1299 cells were treated with C.C, and then cell cycle distribution (A), cell cycle related proteins (B), cell apoptosis (C), apoptotic related proteins (D) and mitotic related proteins (E) were examined.

**Table S1 PCR primers and extension primers of SNPs in 8q22.1-22.2.**

| **SNP ID** | **Forward Primer** | **Reverse Primer** | **Extension primer** |
| --- | --- | --- | --- |
| rs16896067 | 5′-ACGTTGGATGTGGCTGCACAGGCAACAAAAAG-3′ | 5′-ACGTTGGATGTAGGTACAGAGGTAGGATTTG-3′ | 5′-TTCCTGCGTATAAATCTTTGCA-3′ |
| rs62521676 | 5′-ACGTTGGATGGGAACTACCATTAACCTCTTAG-3′ | 5′-ACGTTGGATGCCCACCACAAGACATTTCAAAG-3′ | 5′-ACAAGACATTTCAAAGTAATGAT-3′ |
| rs62521673 | 5′-ACGTTGGATGTCAGAAGAGGCATCATTGTAG-3′ | 5′-ACGTTGGATGGCGTGTAATGTGTCTAGCAAG-3′ | 5′-AGCAAGTACTTGGAAATTATTA-3′ |
| rs10955127 | 5′-ACGTTGGATGGGTGACACTAGAAAAAGTAGTC-3′ | 5′-ACGTTGGATGCCTCTTCATATCTTCCTCACC-3′ | 5′-TGCCTCACCTTATATCTAACCC-3′ |
| rs1136594 | 5′-ACGTTGGATGCGTACAGGAAGAATTCTACAG-3′ | 5′-ACGTTGGATGCTCTGAGCTTGTTTGTTGCTG-3′ | 5′-5′-TTCAACATATGCTTTGCT-3′ |
| rs2449548 | 5′-ACGTTGGATGGCATGAAAGGAGGAATACTGG-3′ | 5′-ACGTTGGATGCATTAAGCCTACAGAATCCCC-3′ | 5′-GGTCCTGTTTGCCACT-3' |
| rs2917959 | 5′-ACGTTGGATGACACAGAGAAAAGCCTCTTAC-3′ | 5′-ACGTTGGATGTGCTGGGATTACAGACATGAG-3′ | 5′-TTTTTGTGGTAGGATATGC-3' |
| rs6468591 | 5′-ACGTTGGATGGAACCAAAAATGTAGGATGGG-3′ | 5′-ACGTTGGATGCCTTCCTCAAAACCTTTATGGC-3′ | 5′-CATTTTCATTAACTATTAGAATTCCT-3' |
| rs2449537 | 5′-ACGTTGGATGTGGGAGTCTGCAGAATTCTGTG-3′ | 5′-ACGTTGGATGCAAAGAGAGTTACAGACCCAG-3′ | 5′-GCAGAACACGTGATTACTGTA-3' |
| rs2513953 | 5′-ACGTTGGATGGGGAGTCCACACTATTTATTG-3′ | 5′-ACGTTGGATGTGCACCACTACCTTTCTATCC-3′ | 5′-AACCCCTACATCCACAC-3' |
| rs2513955 | 5′-ACGTTGGATGGGGATTTTGAAATCCTTTAGTTC-3′ | 5′-ACGTTGGATGTGAAACATGAGTTCACGGGAG-3′ | 5′-ACTCTGTACTTTCATTCCAT-3’ |

**Table S2 PCR primers and extension primers of SNPs in *COX6C*.**

| **SNP ID** | **Forward Primer** | **Reverse Primer** | **Extension primer** |
| --- | --- | --- | --- |
| rs4626565 | 5′-ACGTTGGATGGGAATAGACCTCAGTTGATCCTC-3′ | 5′-ACGTTGGATGTCTGTGAAGAGAAACACAAAGGC-3′ | 5′-AGAGATAAACACTCTCTCAA-3' |
| rs62534633 | 5′-ACGTTGGATGCAATCCCCATGGTAACCACAAAG-3′ | 5′-ACGTTGGATGAGTGACATGTTTCAGTTTCCTTC-3′ | 5′-CTTCTTACTTCCTTATGTGTATATTC-3' |
| rs7812991 | 5′-ACGTTGGATGTCCTCTAATACCTAGAACCAGAC-3′ | 5′-ACGTTGGATGCTAGGACTTCCAGTACTACAATG-3′ | 5′-TGAATAGAAGTGGCAAGAG-3' |
| rs35429753 | 5′-ACGTTGGATGGGCATTATAAGTAGTCCAGAGATG-3′ | 5′-ACGTTGGATGAGCATAGCTGCCCCTCTATCCAT-3′ | 5′-GGAGCCCTGACGATA-3' |
| rs1130474 | 5′-ACGTTGGATGTGGCCATTCTCAAGTTTGTGGCT-3′ | 5′-ACGTTGGATGCATGGTAGTTACTGTCCTTGATAC-3′ | 5′-TGTCCTTGATACGTATGC-3’ |

**Table S3. Summary of the effects COX6C knockdown on mitosis in H1975 cells.**

| **Parameter\Treatment** | **siNC** | **siCOX6C-1** | **siCOX6C-2** |
| --- | --- | --- | --- |
| Mitotic index (%) | 4.32 ± 0.48 | 7.39 ± 1.06 (*) | 7.02 ± 0.26 (**) |
| Prometaphase (%) | 26.15 ± 2.66 | 39.10 ± 2.20 (**) | 32.77 ± 3.08 (*) |
| Telophase (%) | 34.16 ± 4.69 | 14.46 ± 3.14 (**) | 24.12 ± 3.48 (*) |
| Early mitosis (%) | 51.48 ± 4.86 | 81.72 ± 3.23 (***) | 66.10 ± 4.64 (*) |
| Multipolar spindle in Early mitosis cells (%) | 4.32 ± 1.74 | 11.93 ± 1.95 (**) | 8.50 ± 1.32 (*) |
| Lagging chromosome in late mitosis cells (%) | 3.78 ± 1.87 | 35.20 ± 4.85 (***) | 23.63 ± 0.86 (***) |
| Multinuclear cell (%) | 0.49 ± 0.02 | 1.13 ± 0.15 (**) | 0.93 ± 0.12 (**) |

The *P* values were calculated with Student’s *t* test, **P* < 0.05; ***P* < 0.01 and ****P* < 0.001 compare to siNC.
