# Supplementary figures and images for: COX6C expression driven by copy amplification of 8q22.2 regulates cell proliferation via mediation of mitosis by ROS-AMPK signaling in lung adenocarcinoma
Source: Cell Death Dis. 2024 Jan 19;15(1):74. doi: 10.1038/s41419-024-06443-w (PMC10799076; doi:10.1038/s41419-024-06443-w)

**Fig. 2C**

**
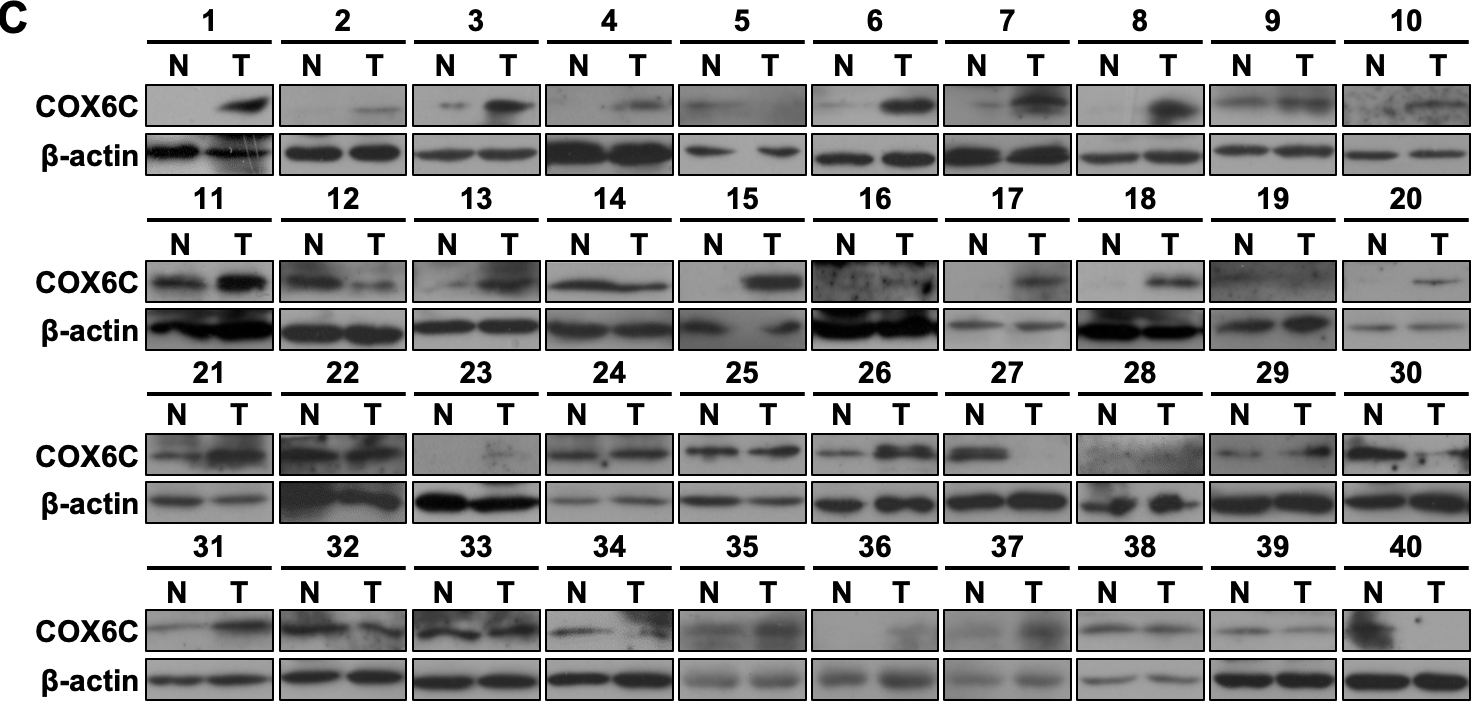
**

**
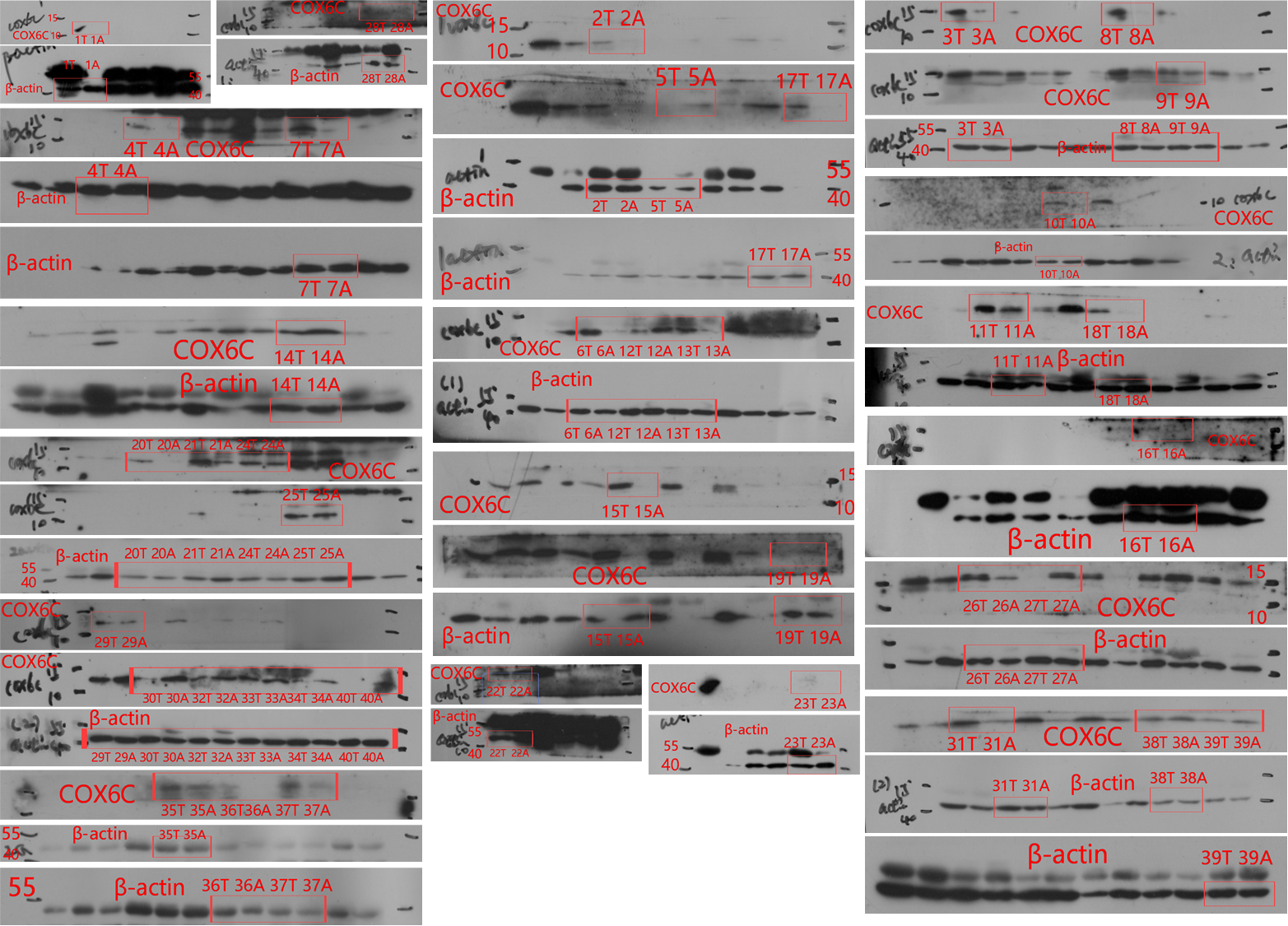
**

**Fig. 2G**

**
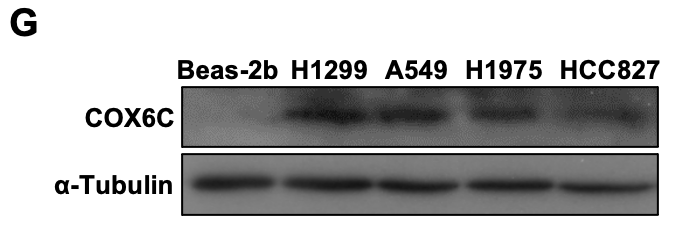
**

**
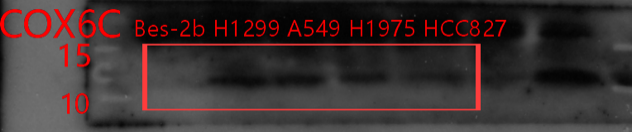

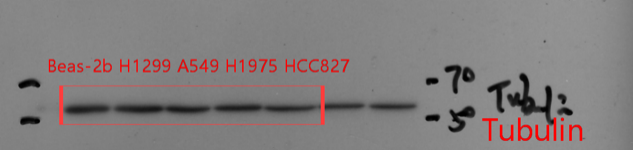
**

**Fig. 3A**


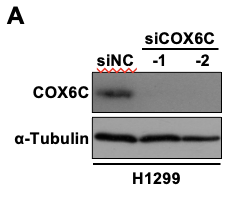


**
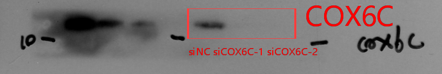

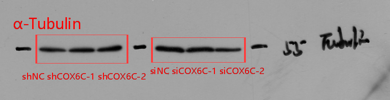
**

**Fig. 3H**

**
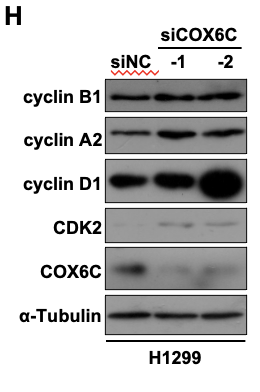

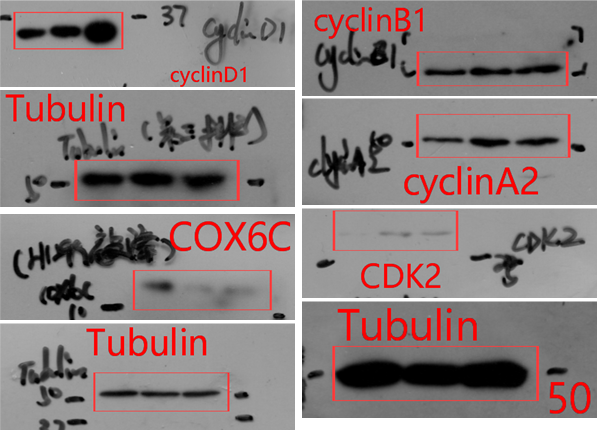
**

**Fig. 3K**

**
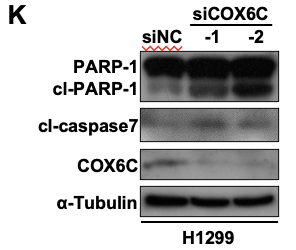

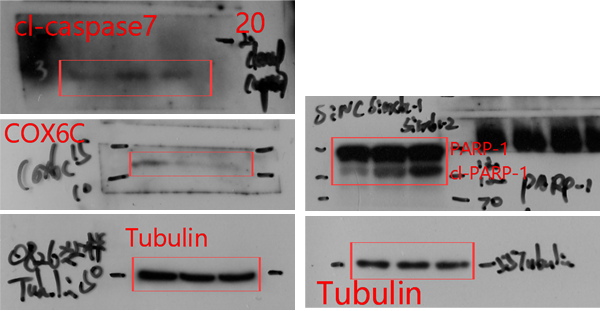
**

**Fig. 4J**

**
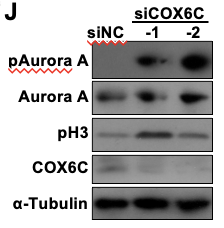

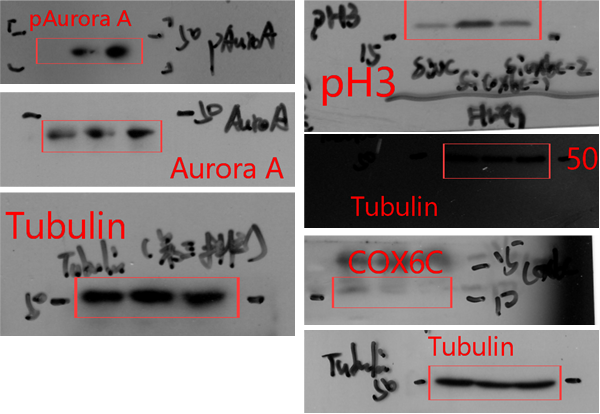
**

**Fig. 5C**

**
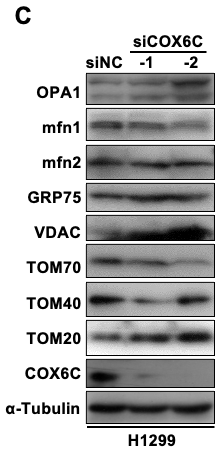

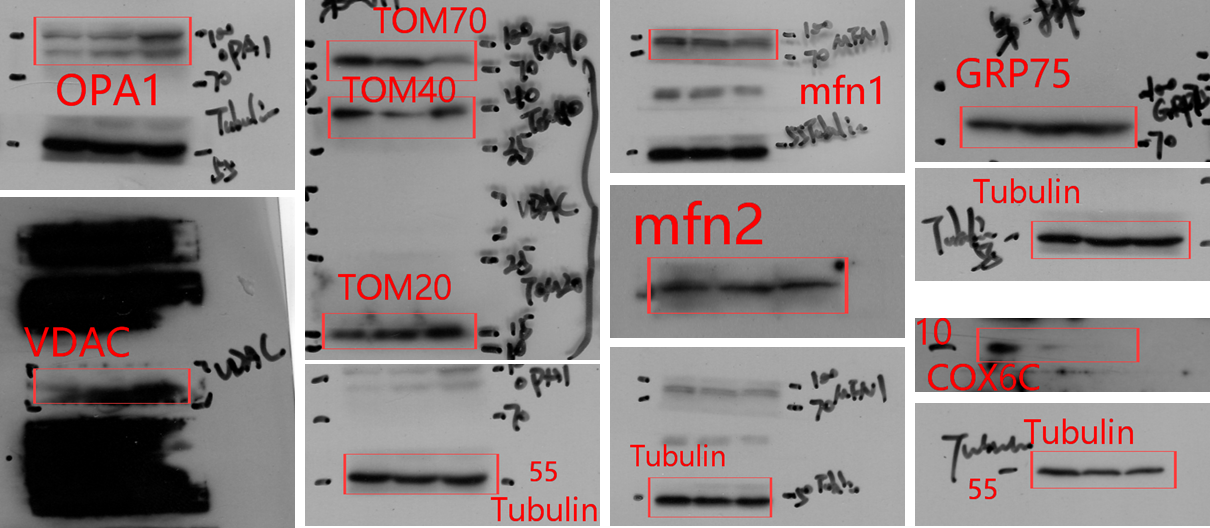
**

**Fig. 6A**


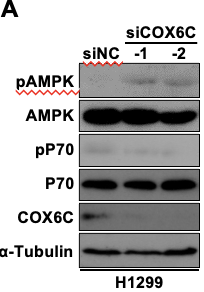
 **
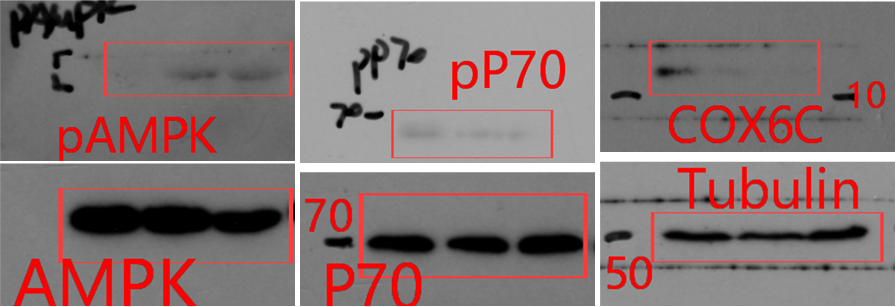
**

**Fig. 6B**

**
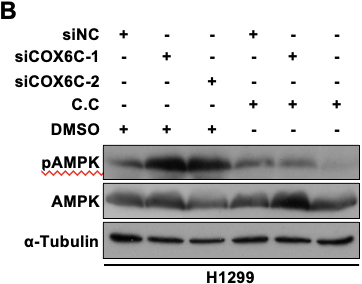
**

**
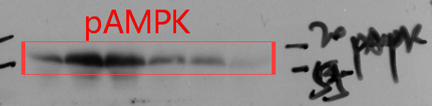

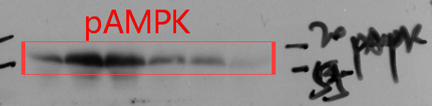

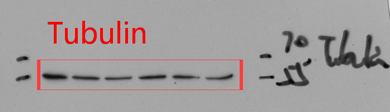
**

**Fig. 7E**

**
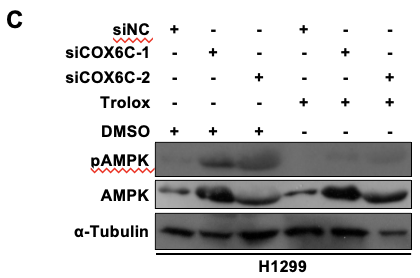
**

**
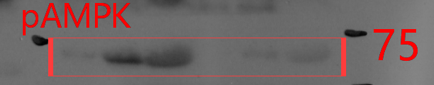

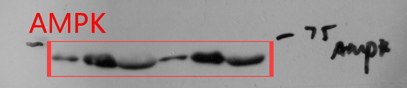

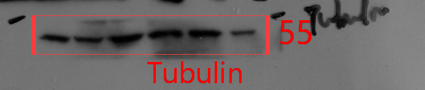
**

**Fig. S2A**

**
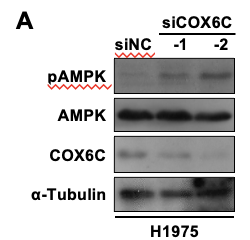
**

**
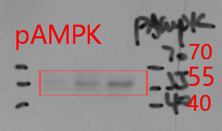

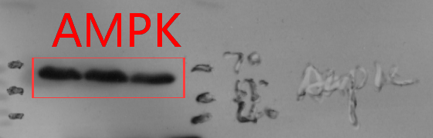

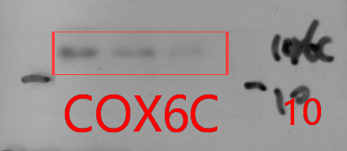

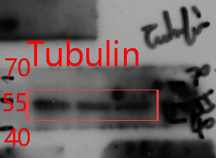
**

**Fig. S3A**


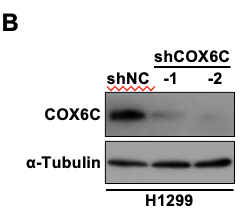


**
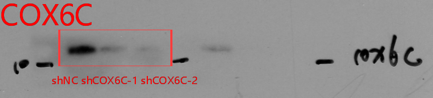

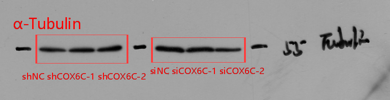
**

**Fig. S6C**

**Fig. S7B**

**
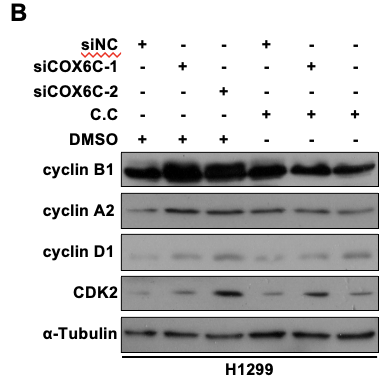

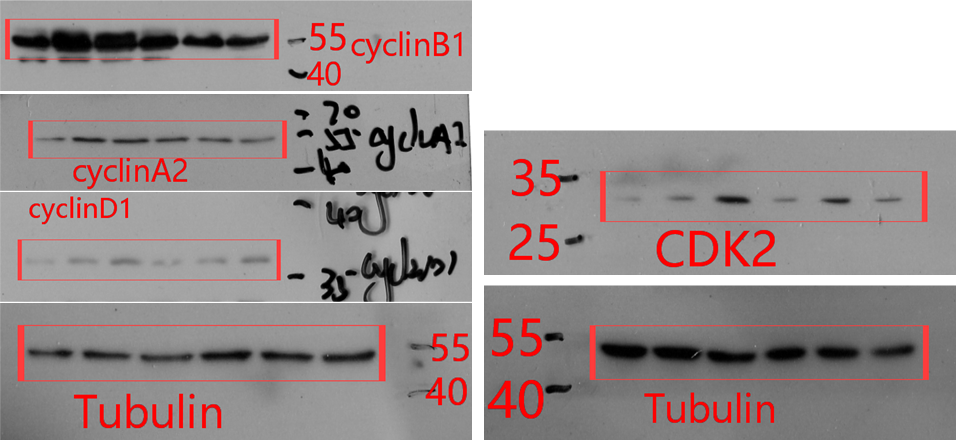
**

**Fig. S7D**

**
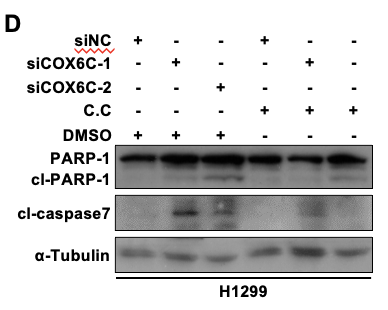

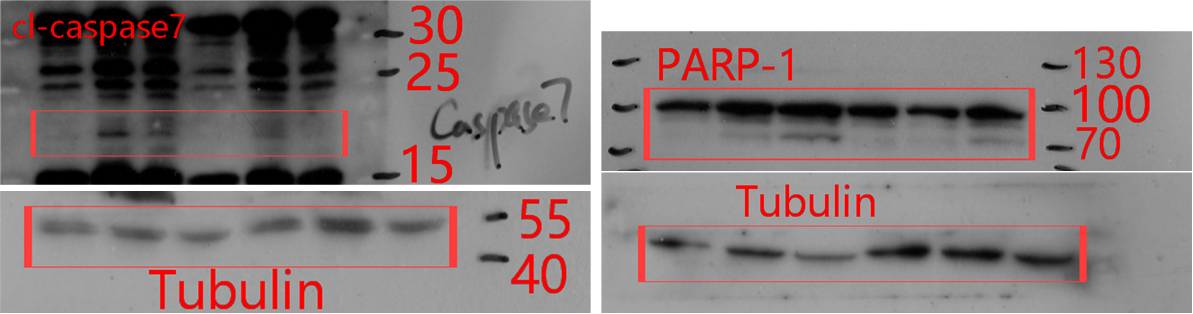
**

**Fig. S7E**

**
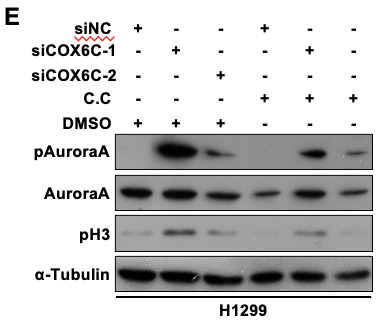

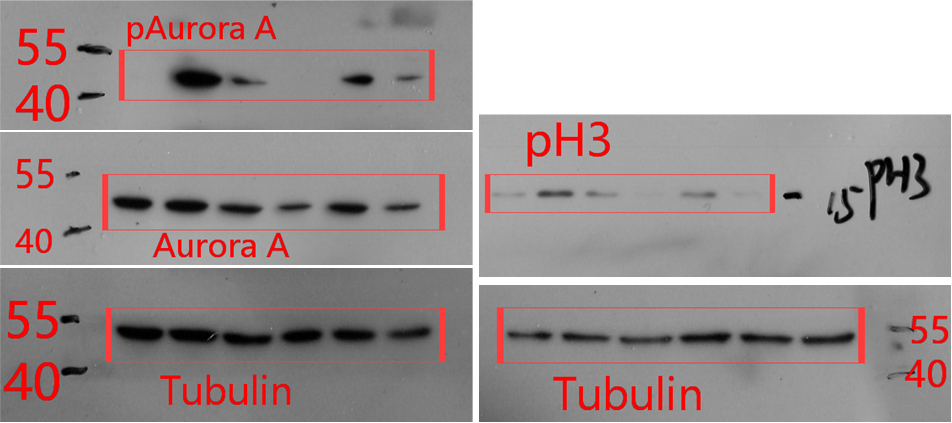
**

Supplement: Supplementary file 3 — Original Data File [file 41419_2024_6443_MOESM3_ESM.docx]
